# The Navigation Guide—Evidence-Based Medicine Meets Environmental Health: Systematic Review of Nonhuman Evidence for PFOA Effects on Fetal Growth

**DOI:** 10.1289/ehp.1307177

**Published:** 2014-06-25

**Authors:** Erica Koustas, Juleen Lam, Patrice Sutton, Paula I. Johnson, Dylan S. Atchley, Saunak Sen, Karen A. Robinson, Daniel A. Axelrad, Tracey J. Woodruff

**Affiliations:** 1 Oak Ridge Institute for Science and Education (ORISE) Postdoctoral Fellow, National Center for Environmental Economics, Office of Policy, U.S. Environmental Protection Agency, Washington, DC, USA; 2 Program on Reproductive Health and the Environment, University of California, San Francisco, Oakland, California, USA; 3 Department of Epidemiology and Biostatistics, University of California, San Francisco, San Francisco, California, USA; 4 Department of Medicine,; 5 Department of Epidemiology, and; 6 Health Policy & Management, Johns Hopkins School of Medicine, Baltimore, Maryland, USA; 7 National Center for Environmental Economics, Office of Policy, U.S. Environmental Protection Agency, Washington, DC, USA

## Abstract

Background: In contrast to current methods of expert-based narrative review, the Navigation Guide is a systematic and transparent method for synthesizing environmental health research from multiple evidence streams. The Navigation Guide was developed to effectively and efficiently translate the available scientific evidence into timely prevention-oriented action.

Objectives: We applied the Navigation Guide systematic review method to answer the question “Does fetal developmental exposure to perfluorooctanoic acid (PFOA) or its salts affect fetal growth in animals ?” and to rate the strength of the experimental animal evidence.

Methods: We conducted a comprehensive search of the literature, applied prespecified criteria to the search results to identify relevant studies, extracted data from studies, obtained additional information from study authors, conducted meta-analyses, and rated the overall quality and strength of the evidence.

Results: Twenty-one studies met the inclusion criteria. From the meta-analysis of eight mouse gavage data sets, we estimated that exposure of pregnant mice to increasing concentrations of PFOA was associated with a change in mean pup birth weight of –0.023 g (95% CI: –0.029, –0.016) per 1-unit increase in dose (milligrams per kilogram body weight per day). The evidence, consisting of 15 mammalian and 6 nonmammalian studies, was rated as “moderate” and “low” quality, respectively.

Conclusion: Based on this first application of the Navigation Guide methodology, we found sufficient evidence that fetal developmental exposure to PFOA reduces fetal growth in animals.

Citation: Koustas E, Lam J, Sutton P, Johnson PI, Atchley DS, Sen S, Robinson KA, Axelrad DA, Woodruff TJ. 2014. The Navigation Guide—evidence-based medicine meets environmental health: systematic review of nonhuman evidence for PFOA effects on fetal growth. Environ Health Perspect 122:1015–1027; http://dx.doi.org/10.1289/ehp.1307177

## Introduction

*Background*. In clinical research, systematic reviews have played a transformative role as a transparent, robust method for synthesizing the available evidence for incorporation into more efficient guidelines and recommendations related to medical interventions. Whereas systematic review methodology has been developed and tested in the clinical sciences for making evidence-based decisions for medical interventions, the methods are not fully transferable to environmental health science, largely because of their primary application to randomized controlled clinical trials, which are, for primarily ethical reasons, unavailable in environmental health. The Navigation Guide was developed to bridge this gap between clinical and environmental health sciences. The methodology provides the capacity to systematically and transparently evaluate the quality and strength of evidence from both human and nonhuman streams of evidence about the relationship between the environment and reproductive and developmental health (Woodruff and Sutton 2014; Woodruff et al. 2011a).

To test and refine the Navigation Guide systematic review methodology, we applied it to the evaluation of experimental animal evidence for the effects of exposure to the environmental contaminant perfluorooctanoic acid (PFOA) on fetal growth. The results of applying the method to the human evidence and integrating the animal and human data into an overarching strength of evidence rating are presented elsewhere (Johnson et al. 2014; Lam et al. 2014).

*Rationale for selecting PFOA.* Environmental exposures to the industrial chemical PFOA are widespread, and PFOA has been detected in the blood of > 95% of the U.S. population [Agency for Toxic Substances and Disease Registry (ATSDR) 2009; Centers for Disease Control and Prevention (CDC) 2009; Kato et al. 2011; U.S. Environmental Protection Agency (EPA) 2009] and in blood samples throughout the world (Kannan et al. 2004; U.S. EPA 2009). Voluntary efforts by eight major manufacturers of PFOA to eliminate global emissions and product content by the end of 2015 are ongoing, and significant progress has been made for both U.S. and non-U.S. operations (U.S. EPA 2008, 2012, 2013a). However, PFOA can remain in the environment, and with a half-life in humans of approximately 3.5 years (Olsen et al. 2007), the chemical will persist in people for years to come (U.S. EPA 2013b, 2014).

Fetal exposure to PFOA may be widespread because the chemical is ubiquitous in the blood of pregnant women and women of child-bearing age and in cord blood (Apelberg et al. 2007a; Calafat et al. 2007; Fei et al. 2007; Midasch et al. 2007; Mondal et al. 2012; Monroy et al. 2008; Woodruff et al. 2011b). The association between PFOA and fetal growth reported in individual human studies has been inconsistent, with some reporting statistically significant associations between prenatal exposure to PFOA and restricted fetal growth (Apelberg et al. 2007b; Fei et al. 2007, 2008) and others reporting no or nonstatistically significant associations (Hamm et al. 2010; Monroy et al. 2008; Washino et al. 2009). The animal literature also includes reports of inconsistent associations between PFOA and fetal growth, including findings of reduced birth weight following prenatal exposure to PFOA in rodents (Butenhoff et al. 2004; Hines et al. 2009; Lau et al. 2004, 2006).

Ubiquitous exposure to a chemical that lacks evidence of nontoxicity is a potential public health concern; moreover, PFOA has been associated with adverse impacts on the quality and duration of the gestation period—one of the most important indicators of an infant’s health and survival (Gluckman and Hanson 2006; Institute of Medicine 2007). Given the potential concern for an adverse developmental health outcome of public health importance and the availability of data, we selected PFOA to test and refine the Navigation Guide method.

## Methods

We assembled a review team to include experts in the fields of risk assessment, environmental health, epidemiology, biology, systematic review, and toxicology to develop a protocol that covered the first three steps of the Navigation Guide systematic review method: *1*) Specify the study question; *2*) select the evidence; and *3*) rate the quality and strength of the evidence (Koustas et al. 2013). Each of the steps of the Navigation Guide method described below involves application of standardized and transparent documentation, including expert judgment. Additional information regarding the Navigation Guide methodology is available elsewhere (Woodruff and Sutton 2014).

### Step 1. Specify the Study Question

Our objective was to answer the question: “Does fetal developmental exposure to PFOA or its salts affect fetal growth in animals?” PICO (participants, interventions, comparator, and outcomes) is an aid used to formulate an answerable question in a systematic review and to provide more specific information about the scope of the review (O’Connor et al. 2011). Because we were evaluating environmental exposures, we used the acronym PECO (i.e., participants, exposure, comparator, and outcomes).

Participants. Animals were from nonhuman species studied during the reproductive/developmental time period (before and/or during pregnancy for females or during development for embryos).

Exposure. Exposure included one or more oral, subcutaneous, or other treatment(s) of any dosage of PFOA (CAS# 335-67-1) or its salts during the time before pregnancy and/or during pregnancy for females or directly to embryos.

Comparator. Experimental animals receiving different doses of PFOA or vehicle-only treatment were used for comparisons.

Outcomes. The outcomes examined for mammalian species were fetal weight near term (e.g., embryonic day 18 for mice and embryonic day 21 for rats) or at birth, and/or other measures of size near term or at birth, such as length. For nonmammalian species, outcomes were weight and/or other measures of size in late stages of embryonic development.

### Step 2. Select the Evidence

*Search methods.* Our search was developed by analyzing the Medical Subject Headings (MeSH) and other terms from the title and abstract text of a group of seven papers known to us, judged to be relevant to our study question, and which represented different journals and years of publication (Abbott et al. 2007; Butenhoff et al. 2004; Hines et al. 2009; Lau et al. 2006; Staples et al. 1984; White et al. 2007, 2009). A list of common and unique terms was compiled and incorporated into a search strategy to address the exposure (PFOA) and outcomes of interest (reproductive/developmental toxicity), as defined in the PECO statement (see Supplemental Material, Tables S1, S2). To develop search terms to retrieve experimental animal studies, we adapted a search filter developed by Hooijmans et al. (2010b).

We searched PubMed (http://www.ncbi.nlm.nih.gov/pubmed) and Web of Science (http://apps.webofknowledge.com/) on 3 February 2012. Using PFOA terms, we searched 35 toxicological databases between 23 January and 6 February 2012. Our search was not limited by language or publication date. We hand searched the reference list of all included studies and searched for publications citing the included studies. We also consulted with a subject matter expert (C. Lau, U.S. EPA).

*Data collection and management.* We imported or manually entered all retrieved records into EndNote X4 reference management software (http://thomsonreuters.com/endnote/), and each record was assigned a source identification number, which was used to track individual studies throughout the course of the review. Two authors (E.K. and J.L.) independently screened the titles and abstracts of each record retrieved to identify those meeting our inclusion criteria using DistillerSR (Evidence Partners; http://www.systematic-review.net). We developed inclusion and exclusion criteria based on our PECO statement. All studies that compared experimental animals exposed to one or more doses of PFOA during reproductive or developmental periods with untreated control experimental animals were eligible for inclusion. We excluded studies if one or more of the following criteria were met: the article did not contain original data (i.e., review article); study subjects were not animals; PFOA was not administered to study subjects; and PFOA was not administered during the reproductive/developmental time period. Two authors (E.K. and J.L.) assessed the full text of studies that could not be excluded based on screening of the title and abstract. Potentially relevant non-English articles were translated to determine eligibility. To provide quality control, a third author (P.I.J.) screened the title and abstract of 5% of the search results and 5% or five articles—whichever was greater—of the search results eligible for full text. We considered studies that described more than one experiment or outcome measure as separate data sets.

Two authors (E.K. and J.L.) independently extracted data relating to study characteristics and outcome measures from all included articles into a Microsoft Access 2007 database. The list of extracted study characteristics was based on a compilation of previously published checklists and criteria (Guyatt et al. 2011; Higgins and Deeks 2011; Hooijmans et al. 2010a; Kilkenny et al. 2010).

One author (E.K.) performed data entry of the raw outcome data using Microsoft Excel 2007, and a second author (D.S.A.) verified all values. We contacted study authors when additional information was required for performing statistical analysis and/or analysis of the full data set. For example, we requested numerical estimates associated with figures presented in published articles, numbers of animals allocated to various test groups, and raw data values. In some cases, fetal growth data were not presented in the published study because the outcome was not of primary interest to the study authors. If there was a reason to believe the study authors may have measured fetal growth, we contacted them to obtain any data they may have collected during the course of the study. We also contacted all study authors to inform them of our systematic review and to verify values used in both the meta-analysis and analysis of the full data set.

*Statistical analyses.* Two authors (E.K. and J.L.) assessed study characteristics from all included articles for comparability (i.e., study features and biological heterogeneity) to determine which studies were suitable for meta-analysis. We consulted experts in the field of PFOA toxicity, toxicological study design, or human/animal toxicity reviews to develop these characteristics and their associated heterogeneity concerns beforehand. For example, we considered the differences in PFOA clearance rates between female mice (approximately 17 days) and rats (2–4 hr) as a potential biological heterogeneity concern (Lau et al. 2007).

From the assessment of specified characteristics, we determined that only a subset of data was combinable in a meta-analysis. This subset of seven studies (eight data sets) had the following characteristics:

Species: mouse
Route of exposure: gavage
Method of outcome measurement: weight
Time point of outcome measurement: at or soon after birth.

We used the mean (± SE) pup body weight at birth from each of the eight data sets for all PFOA doses < 5 mg/kg body weight (mg/kg BW)/day. We limited the dose range in order to focus on effects at lower tested doses and to minimize adverse impacts from responses at higher doses (such as litter loss) on the overall estimate. We used a two-step modeling approach. In the first step, we analyzed each data set separately using a linear mixed-effects model, and we obtained a slope estimate of the dose–response effect (and the associated SE). In the second step, we combined the slope and SE estimate from each data set using a random-effects model.

The result was an estimate of the overall mean change in body weight per offspring for a 1-unit increase in mg/kg BW/day dose, accounting for within- and between-study variability. We used the programming environment R, version 2.13.1 (R Development Core Team; http://www.R-project.org/) and its standard packages. We used the metafor package in R (Viechtbauer 2010) for conducting our random effects meta-analysis.

To visually assess the possibility of publication bias in a meta-analysis, we considered producing a funnel plot of the estimated effects. However, tests for funnel plot asymmetry are not recommended when there are < 10 studies because test power is usually too low to distinguish chance from real asymmetry (Sterne et al. 2011a). Because our meta-analysis was limited to 7 studies (8 data sets), we did not produce a funnel plot.

*Statistical heterogeneity assessment.* We sought to assess whether differences in estimated effect sizes among studies were consistent with random variation versus nonrandom heterogeneity among the studies. We estimated the between-study variance component and tested the null hypothesis that the between-study variability was absent using Cochran’s *Q* statistic. The test statistic follows a chi-square distribution with *n* – 1 degrees of freedom, where *n* is the number of studies. We considered *p* ≤ 0.05 statistically significant. We also calculated the *I*^2^ statistic, which estimates the percentage of variation across studies due to heterogeneity rather than chance (Higgins et al. 2003), and used the Cochrane Collaboration’s guidelines to interpret the statistic, where a value of > 50% may indicate substantial heterogeneity (Deeks et al. 2011). To assess the overall impact of existing study heterogeneity on the meta-analysis, we considered the magnitude/direction of effect estimates, the *I*^2^ statistic, and the *p*-value from the Cochran’s *Q* test.

*Sensitivity analysis.* We performed sensitivity analyses using subgroup analyses based on characteristics described above that were used to determine comparability across studies for the meta-analysis. To evaluate the influence of each individual study on the main meta-analysis results and assist in identifying any study characteristics that might be influential in the final results, we performed a sensitivity analysis by removing one data set at a time from the meta-analysis.

*Analysis of the full data set.* We analyzed all included animal studies identified via our search and exclusion/inclusion assessment to assess the totality of all available animal evidence. This was done to maximize use of all data, in addition to those determined appropriate to combine in the meta-analysis. To assess results from the full data set, we calculated the percentage change in outcome (weight or length) compared with the control group for each tested dose group for each of the data sets and used these values to create scatter plots. Two of the nonmammalian studies reported outcome measurements at multiple time points during larval development (Spachmo and Arukwe 2012; Wang et al. 2010). We selected the outcome measurement reported at the latest time point during the larval stage, based on justification that this allowed for consideration of maximal larval growth. For each study, we used the mean and SE estimates reported by authors to calculate a 95% confidence interval (CI) for the difference in means comparing each treatment group with the control group. We interpreted a 95% CI that overlapped zero as indicating no statistically significant difference between the mean weight in that treatment group with the mean weight in the control group.

### Step 3. Rate the Quality and Strength of the Evidence

**Figure 1:**
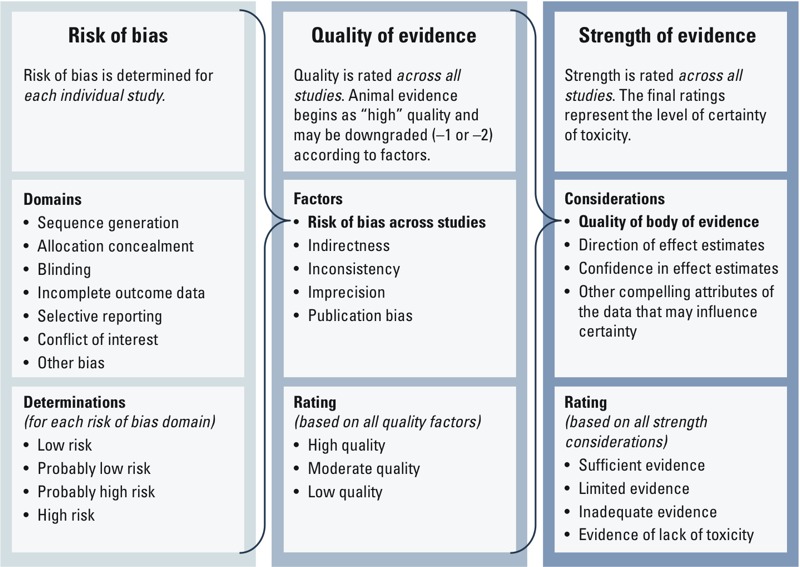
Flowchart for evaluating risk of bias, quality of evidence, and strength of evidence.

To rate the evidence, we *a*) determined the risk of bias for individual studies based on seven domains; *b*) rated the overall quality across all studies in the body of evidence based on five factors, including risk of bias; and *c*) rated the overall strength of evidence across all studies in the body of evidence based on four considerations, including the quality of the body of evidence (Figure 1).

Assessment of risk of bias. Two authors (E.K. and J.L.) assessed risk of bias defined as characteristics of a study that can introduce a systematic error in the magnitude or direction of the results of the study (Higgins et al. 2011) for included studies based on seven risk of bias domains using modified terminology and concepts in the Cochrane Collaboration’s Tool for Assessing Risk of Bias. Informed by empirical data from meta-analyses conducted on pharmacological treatments (Roseman et al. 2011), we considered funding source and reported conflicts of interest to be potential sources of bias. We did not ask study authors for additional information to inform our risk of bias determinations. However, if study authors mentioned study design details in their responses to our requests for data, we considered the information while evaluating risk of bias. See Table 1 for a summary of the risk of bias domains assessed for each included study (see also Supplemental Material, “Instructions for making risk of bias determinations”).

**Table 1:** Tool for assessing risk of bias.

Domain	Criteria for low risk of bias rating	Examples of factors considered in assessment
Sequence generation	Study authors reported the use of a random component in the sequence generation process.	Use of a random component, such as a random number table or computer random number generation; statement by study author that animals were randomly allocated.
Allocation concealment	Study authors reported that study personnel could not foresee which animals were allocated to the various experimental groups.	Use of sequentially numbered cages or animals.
Blinding	Study authors reported that personnel and outcome assessors were adequately prevented from knowledge of the allocated exposures during the study.	Use of masked identifiers in the study and for outcome assessment.
Incomplete outcome data	Study authors reported when and why participants left the study.	The number of animals allocated to experimental groups was reported and/or adequate follow up of dams and offspring (for mammalian studies) was carried out; the number of organisms allocated to experimental groups was reported and/or organisms were adequately followed up after exposure (for non­mammalian studies).
Selective reporting	The study’s prespecified outcomes that are of interest in the review were reported in a prespecified way.	The number of animals or organisms analyzed for outcomes of interest was reported, or study authors provided additional data; study methods matched study results for outcomes of interest.
Conflict of interest	The study was free of support from a company, study author, or other entity having a financial interest in the exposures of interest in the review.	The study was funded or conducted by companies with a financial interest in PFOA; companies provided services to assist in the completion of the study, evaluate the data, or write the manuscript; or the publication or report included a declaration of conflicts of interest.
Other bias	Study appears to be free of other sources of bias.	Other potential sources of bias related to the specific study design.

*Rate the quality and strength of the body of evidence.* Upon completion of the data analysis, each of the nine review authors compared the results of the systematic review to the Navigation Guide factors and considerations for rating the quality and strength of the nonhuman evidence. The Navigation Guide rating method (Woodruff et al. 2011a) was applied according to explicit written directions (Koustas et al. 2013). Because of fundamental biological differences between mammalian and nonmammalian model systems, we evaluated the mammalian and nonmammalian studies as separate bodies of evidence.

The possible ratings for the overall quality of the body of evidence were “high,” “moderate,” and “low.” These quality ratings were determined by assigning an initial rating according to the type of study, and then downgrading the rating if factors that decrease the quality level of the studies were present. The initial quality rating assigned to both the mammalian and nonmammalian bodies of evidence was “high,” comparable to the rating assigned to human experimental studies [i.e., randomized controlled trials (RCTs)] in systematic review methods used in the clinical sciences. An initial “high” quality rating for experimental animal studies was supported by the level of study control exercised in such studies and the limited heterogeneity within an experimental animal study population. This is also consistent with Grading of Recommendations Assessment, Development and Evaluation (GRADE) guidelines for clinical evidence that consider randomization a key determinant of a “high” grade (Guyatt et al. 2011). Upgrades to the quality rating for experimental animal data were not considered because the initial quality level was “high.”

The overall body of evidence was evaluated for downgrading based on the presence of five factors (Figure 1):

Risk of bias across studies: a substantial risk of bias existed across the body of evidence.
Indirectness: Evidence was not directly comparable to the question of interest (i.e., population, exposure, comparator, and/or outcome). Beforehand, we decided not to downgrade experimental animal studies for indirectness because studies find that humans are as sensitive or more sensitive to chemical exposures than animals, strengthening the applicability of findings from experimental animal studies to human health outcomes (Kimmel et al. 1984; U.S. EPA 1996). However, in applying GRADE principles to the Navigation Guide, evidence would be rated down if the animal model was determined to be biologically inappropriate for the health outcome under study.
Inconsistency: Estimates of effect were widely different (heterogeneity or variability in results).
Imprecision: Studies had few participants and few events (wide CIs).
Publication bias: Studies were missing from the body of evidence, resulting in an overestimate or underestimate of true effects from exposure.

According to GRADE, these five factors address nearly all issues that bear on the quality of evidence (Balshem et al. 2011). Each of the nine review authors reviewed the body of evidence and applied their expert judgment to independently and transparently grade the quality of evidence based on the presence of the five objective factors using detailed instructions (Koustas et al. 2013). Possible ratings were 0 (no change from “high” quality), –1 (one-level downgrade to “moderate” quality) or –2 (two-level downgrade to “low” quality). Consistent with GRADE’s approach to evaluating risk of bias across studies (Guyatt et al. 2011), authors were instructed to be conservative in making judgments to downgrade the evidence for all factors (i.e., high confidence of substantial concerns with the body of evidence before rating down). Authors reviewed the body of evidence as a way to initiate the group discussion and gather all perspectives for consideration. After independently evaluating the quality of the evidence, all authors discussed their evaluations. The discussion between coauthors was extensive and iterative and was carried out over several meetings until a consensus was reached. These collective decisions did not involve a “majority vote” or other tallying of perspectives. It was prespecified that discrepancies between review authors that could not be resolved through consensus would be resolved by the senior author (T.J.W.). However, for this case study, review authors were able to agree on a collective consensus for each rating and the arbiter was not necessary. The rationale for each collective decision on each of the five factors was recorded.

In systematic reviews in the clinical sciences, rating the quality of evidence is the final step because only one stream of evidence is considered in a decision. However, given that our purpose was to ultimately integrate the strength of multiple streams of evidence used in environmental health decision making (i.e., toxicology and epidemiology) leading to a concise “bottom line” statement about a chemical’s toxicity that brings all of the relevant evidence to bear, the Navigation Guide systematic review method specifies an additional step: moving from quality of evidence to strength of evidence.

We rated the overall strength of the evidence based on a combination of four considerations: *a*) quality of the body of evidence, *b*) direction of effect estimates, *c*) confidence in effect estimates (likelihood that a new study would change our conclusion), and *d*) other compelling attributes of the data that may influence certainty (Figure 1). The results of rating the strength of the nonhuman evidence were compared with the definitions specified in the Navigation Guide for “sufficient” evidence of toxicity; “limited” evidence of toxicity; “inadequate” evidence of toxicity; or “evidence of lack of toxicity” (Table 2), which were based on criteria in use by the International Agency for Research on Cancer (IARC 2006) and the U.S. EPA (1991, 1996). The procedure for rating the strength of the evidence was similar to rating the quality of evidence: All review authors independently evaluated the strength of the evidence according to the same four considerations, and then they compared their evaluations, resolved any discrepancies through discussion, and recorded the rationale for every collective decision.

**Table 2:** Strength of evidence definitions for non­human studies.^*a*^

Strength rating	Definition
Sufficient evidence of toxicity	A positive relationship is observed between exposure and adverse outcome in multiple studies or a single appropriate study in a single species.^*b*^ The available evidence includes results from one or more well-designed, well-conducted studies, and the conclusion is unlikely to be strongly affected by the results of future studies.^*c*^
Limited evidence of toxicity	The data suggest a positive relationship between exposure and adverse outcome, but there are important limitations in the quality of the body of evidence. Confidence in the relationship is constrained by factors such as the number, size, or quality of individual studies, or inconsistency of findings across individual studies.^*c*^ As more information becomes available, the observed effect could change, and this change may be large enough to alter the conclusion.
Inadequate evidence of toxicity	The available evidence is insufficient to assess effects of the exposure. Evidence is insufficient because of the limited number or size of studies, low quality of individual studies, or inconsistency of findings across individual studies. More information may allow an assessment of effects.
Evidence of lack of toxicity	Data on an adequate array of end points from more than one study with at least two species showed no adverse effects at doses that were minimally toxic in terms of inducing an adverse effect. Information on pharmacokinetics, mechanisms, or known properties of the chemical class may also strengthen the evidence.^*d*^ The conclusion is limited to the species, age at exposure, and/or other conditions and levels of exposure studied, and is unlikely to be strongly affected by the results of future studies.^*c*^
^***a***^The Navigation Guide rates the quality and strength of evidence of human and non­human evidence streams separately as “sufficient,” “limited,” “inadequate,” or “evidence of lack of toxicity,” and then these two ratings are combined to produce one of five possible statements about the overall strength of the evidence of a chemical’s reproductive/developmental toxicity. The methodology is adapted from the criteria used by IARC to categorize the carcinogenicity of substances (IARC 2006) except as noted. ^***b***^IARC’s criteria for sufficient evidence of carcinogenicity in animals requires multiple positive results (species, studies, sexes) (IARC 2006). The Navigation Guide integrates the U.S. EPA’s minimum criteria for animal data for a reproductive or developmental hazard (i.e., data demonstrating an adverse reproductive effect in a single appropriate, well-executed study in a single test species) (U.S. EPA 1996). The Navigation Guide also incorporates the U.S. EPA’s ”sufficient evidence category,” which includes data that “collectively provide enough information to judge whether or not a reproductive hazard exists within the context of effect as well as dose, duration, timing, and route of exposure. This category may include both human and experimental animal evidence” (U.S. EPA 1996). The U.S. EPA statement for developmental hazards is slightly different but includes the same relevant information regarding dose, duration, timing, and so on (U.S. EPA 1991). ^***c***^Language for the definitions of the rating categories were adapted from descriptions of levels of certainty provided by the U.S. Preventive Services Task Force Levels of Certainty Regarding Net Benefit (Sawaya et al. 2007). ^***d***^Based on minimum data requirements according to U.S. EPA guidelines for assessing reproductive toxicity (U.S. EPA 1996).	

## Results

### Included Studies

**Figure 2:**
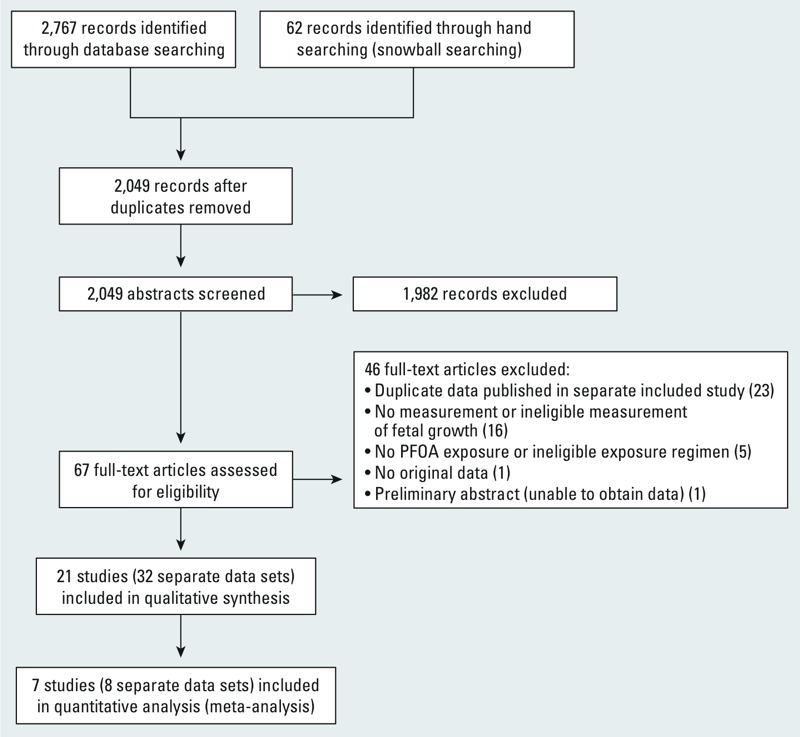
Flowchart of the study-selection process.

Of the 2,049 unique records we identified (see Supplemental Material, Table S3 for the total number of hits retrieved from each database), 1,982 were excluded through title and abstract screening and 46 articles were excluded during full-text review, resulting in 21 studies describing 32 data sets included in the review (Figure 2). A summary of mammalian and nonmammalian study characteristics are provided in Tables 3 and 4, respectively. Detailed characteristics of each mammalian and nonmammalian study are provided in Supplemental Material, Tables S4–18 and Tables S19–S24, respectively. Various details of outcome data and study design characteristics necessary for data analysis were missing from all 21 articles. In some cases, published articles did not include details needed for our analysis, such as numerical outcome measurements or data on fetal growth, if this was not a primary outcome of interest for study authors. In other cases basic information, such as allocation numbers or the number of animals weighed to obtain given outcome values, was missing. Our efforts to contact study authors resulted in obtaining additional data for 18 of the 21 included studies, along with raw data in many instances (see Supplemental Material, Tables S4–S24).

**Table 3:** Summary of mammalian study characteristics.

Source [source ID]	Species	Time point of outcome measurement	Outcome measure	Route of exposure	Period of exposure	PFOA dose range (mg/kg BW/day)^*a*^	No. of doses administered^*b*^	No. of litters	Reason(s) excluded from meta-analysis
Studies used in meta-analysis
Hines et al. 2009 [260]	Mouse	Birth	Weight	Gavage	GDs 1–17	0.01–5	5	75	NA
White et al. 2009 [312]	Mouse	Birth	Weight	Gavage	GDs 8–17	5	1	8	NA
Abbott et al. 2007 [528]	Mouse	Birth	Weight	Gavage	GDs 1–17	0.1–1	4	58	NA
White et al. 2007 [566]	Mouse	Birth	Weight	Gavage	GDs 1–17 GDs 8–17 GDs 12–17	5	1	37	NA
Wolf et al. 2007 [571]	Mouse	Birth	Weight	Gavage	GDs 1–17	3–5	2	87	NA
Wolf et al. 2007 [571]	Mouse	Birth	Weight	Gavage	GDs 7–17 GDs 10–17 GDs 13–17 GDs 15–17	5–20	2	56	NA
Lau et al. 2006 [635]^*c*^	Mouse	Birth	Weight	Gavage	GDs 1–17	1–20	5	103	NA
White et al. 2011 [3862]	Mouse	Birth	Weight	Gavage	GDs 1–17	1–5	2	60	NA
Studies not used in meta-analysis
Hu et al. 2010 [68]	Mouse	Birth	Weight	Drinking water	GDs 6–17	0.05–1	2	30	Incomparable route of exposure
Yahia et al. 2010 [103]	Mouse	Fetal	Weight	Gavage	GDs 0–17	1–10	3	29	Incomparable time point of outcome measurement
Yahia et al. 2010 [103]	Mouse	Birth	Weight	Gavage	GDs 0–18	1–10	3	20	Time point of birth weight measurement was not specified
Fenton et al. 2009 [264]	Mouse	Fetal	Weight	Gavage	GD17	0.1–5	3	19	Incomparable time point of outcome measurement
Fenton et al. 2009 [264]	Mouse	Birth	Weight	Gavage	GD17	0.1–5	3	19	Dams were exposed for only 1 day of pregnancy
Lau et al. 2006 [635]^*c*^	Mouse	Fetal	Weight	Gavage	GDs 1–17	1–40	6	183	Incomparable time point of outcome measurement
Hinderliter et al. 2005 [711]^*d*^	Rat	Birth	Weight	Gavage	GDs 4–21	3–30	3	20	Incomparable species
Staples et al. 1984 [1871]	Rat	Fetal	Weight	Gavage	GDs 6–15	100	1	46	Incomparable species and time point of outcome measurement
Staples et al. 1984 [1871]	Rat	Fetal	Weight	Inhalation	GDs 6–15	0.1–25 mg/m^3^	4^*e*^	103	Incomparable species, route of exposure, and time point of outcome measurement
Staples et al. 1984 [1871]	Rat	Birth	Weight	Gavage	GDs 6–15	100	1	21	Incomparable species
Staples et al. 1984 [1871]	Rat	Birth	Weight	Inhalation	GDs 6–15	0.1–25 mg/m^3^	4	54	Incomparable species and route of exposure
Boberg et al. 2008 [3061]	Rat	Fetal	Weight	Gavage	GDs 7–20/21	20	1	11	Incomparable species and time point of outcome measurement
Onishchenko et al. 2011 [3610]	Mouse	Birth	Weight	Food	GDs 1–20	0.3	1	15	Incomparable route of exposure
York 2002 [5122]^*f*^	Rat	Birth	Weight	Gavage	70 days prior to breeding throughout lactation	1–30	4	141	Incomparable species
GD, gestation day. ^***a***^Unless otherwise specified; the dose range is limited to those doses for which dams were analyzed. ^***b***^Excludes control groups; study used one control group unless otherwise specified. ^***c***^Lau (2006) is listed two times (birth weight data were included in meta-analysis; fetal data were excluded from meta-analysis). ^***d***^Hinderliter (2005) is a peer-reviewed publication; the author provided an industry report with detailed data (Mylchreest 2003). ^***e***^Included three control groups. ^***f***^York (2002) is an industry report; the search also identified peer-reviewed journal publications describing findings from the report (Butenhoff et al. 2004; York et al. 2010), but these journal publications were excluded as duplicates because the report provided raw data.									

**Table 4:** Summary of non­mammalian study characteristics.

Source [source ID]	Species	Time point(s) of outcome measurement	Outcome measure	Route of exposure	Period of exposure	PFOA dose range	No. of doses administered^*a*^	No. of offspring
Hagenaars et al. 2011 [59]	Zebrafish	120 hpf (posthatching)	Length	Egg immersion	Spawning, 120 hpf	15–250 mg/L	8	292
Wang et al. 2010 [86]	Fruit fly	30, 48, 72, 96, and 110 ael (larval stages)	Length^*b*^	Food	Egg laying, 110 ael	100–500 μM	2	378
Wang et al. 2010 [86]	Fruit fly	Pupae	Weight	Food	Egg laying, white pupae stage	100–500 μM	2	98
Pinkas et al. 2010 [187]	Chicken	Hatchling	Weight	Egg injection	Single treatment at incubation day 0	5–10 mg/kg egg	2	52
O’Brien et al. 2009 [236]	Chicken	Embryo at pipping star or day 22, whichever came first	Weight	Egg injection	Single treatment at incubation day 0	0.01–10 mg/kg egg	4^*c*^	37
Jiang et al. 2012 [3926]	Chicken	Embryonic day 19	Yolk-free body weight	Egg injection	Single treatment at incubation day 0	0.5–2 mg/kg egg	2^*c*^	40
Jiang et al. 2012 [3926]	Chicken	16–24 hr posthatching	Yolk-free body weight and crown to rump length	Egg injection	Single treatment at incubation day 0	0.5–2 mg/kg egg	2^*c*^	68
Spachmo and Arukwe 2012 [3932]	Salmon	Study days 21, 35, 49, 56 (larval stages posthatching)	Length and dry weight	Egg immersion	Egg stage, day 48	100 μg/L	1	80
Abbreviations: ael, hours after egg laying; hpf, hours postfertilization. ^***a***^Excludes control groups; study used one control group unless otherwise specified. ^***b***^Length measurements provided by study author (used to calculate volume outcome reported in study). ^***c***^Included two control groups.								

*Populations*. Of the 21 studies, 15 were conducted on mammalian species (11 mouse and 4 rat) and 6 studies were conducted on nonmammalian species (3 chicken, 1 fruit fly, 1 zebrafish, and 1 salmon) (Tables 3 and 4).

*Mammalian exposures*. For all 15 mammalian studies, pregnant female dams were exposed to PFOA, and fetal growth was measured in the resulting progeny (Table 3). The primary route of exposure was oral gavage (13 studies), but some studies also evaluated exposures via inhalation, food, and water. Most of the mammalian studies (12) exposed dams to the ammonium salt form of PFOA (CAS# 3825-26-1), 1 study exposed dams to the free acid form (CAS# 335-67-1), and 2 studies did not specify the form used for exposure. The dose range tested varied widely across studies, ranging from 0.01 to 100 mg/kg BW/day. Inhalation study doses ranged between 0.1 and 25 mg/m^3^. The number of PFOA doses administered per study ranged from one to six. Although dams in all studies were exposed to PFOA at some point during their pregnancy, the window of exposure varied across studies from a single gavage exposure on a single day of pregnancy to exposure prior to conception that continued throughout pregnancy.

*Mammalian comparators*. Eleven gavage studies used water as a vehicle control and two used corn oil (Table 3). The inhalation study utilized three control groups: in-house air only, in-house air pair-fed 10 mg/m^3^ PFOA, and in-house air pair-fed 25 mg/m^3^ PFOA (Staples et al. 1984). The PFOA-treated food study (Onishchenko et al. 2011) used ethanol-treated food as a control, and the PFOA-treated water study (Hu et al. 2010) used untreated water as a control. Other than PFOA exposure, all control groups were treated similarly to dose groups for each data set.

*Mammalian outcomes*. Body weight was used as the outcome measure for all 15 mammalian studies (Table 3). Because pregnant dams were exposed to PFOA for all mammalian studies, the litter was used as the statistical unit; the total number analyzed across studies ranged from 8 to 183 litters.

The time point of weight measurement varied between fetal time points near term, typically gestation day (GD) 18 for mice and GD21 for rats, to at or near the time of birth, typically postnatal day (PND) 0 to PND2. The methods used to monitor parturition varied widely across birth weight studies, from constant monitoring to daily cage checks. PND1 was defined as either the day of birth or the day after birth.

The method of weight measurement varied across studies as well, from weighing offspring individually, grouped by litter, or grouped by sex, to weighing a subset of offspring from each litter. Offspring survival was statistically significantly reduced (based on the alpha level specified by study authors, generally < 0.05 or ≤ 0.05) at exposure to doses > 5 mg/kg BW/day in five studies; one study did not provide statistics or comment on litter sizes at birth.

**Figure 3:**
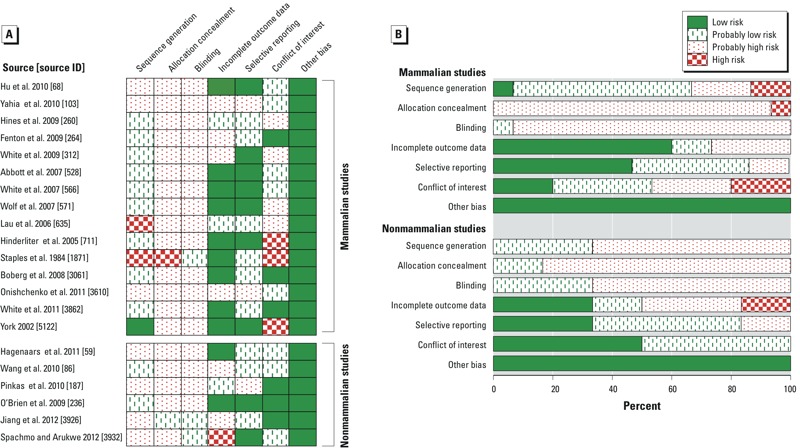
Summary of review authors’ risk of bias judgments (low, probably low, probably high, and high risk) for each risk of bias item for each included study (*A*) and given as percentages across all included studies (*B*), separated into mammalian (*n = *15) and non­mammalian (*n = *6) studies.

*Mammalian risk of bias assessment*. On the basis of our risk of bias assessment, we concluded that the majority of studies had “probably high” risk of bias for allocation concealment and blinding, and “probably low” risk of bias for incomplete outcome data and selective reporting. Ratings for sequence generation and conflict of interest were mixed across studies, and ranged from low to high risk of bias. All studies had low risk of bias for the “other bias” domain (Figure 3A,B). See Supplemental Material, Tables S25–S39, for details on the risk of bias results for each mammalian study.

*Nonmammalian exposures*. Developing embryos were directly exposed to PFOA in all six nonmammalian studies (Table 4). Routes of administration varied based on test species: injection of PFOA solution into eggs for chickens, immersion of eggs in PFOA solution for zebrafish and salmon, and PFOA-treated food for fruit flies. One study exposed organisms to the ammonium salt form of PFOA (CAS#3825-26-1), two studies exposed organisms to the free acid form of PFOA (CAS#335-67-1), and three studies did not specify the form of PFOA. The dose ranges across studies varied based on animal species tested: chicken (0.01–10 mg/kg egg), zebrafish (15–250 mg/L water), fruit fly (100–500 μM in food), and salmon (100 μg/L water). The number of PFOA doses administered per study ranged from one to eight.

In all nonmammalian studies, embryos were exposed during development, and the time period of exposure varied based on species. For the chicken studies, a single injection of PFOA was administered to eggs on incubation day 0; for zebrafish studies, eggs were exposed from 60 min after spawning to 120 hours post fertilization (hpf); for salmon studies, eggs and larvae were exposed to PFOA-containing water for 48 days; for fruit fly studies, female flies were placed in vials with PFOA-containing food and allowed to lay eggs for a period of 2 hr, and eggs were allowed to hatch and develop through 110 hr after egg laying (ael) or to the white pupae stage, depending on data set.

*Nonmammalian comparators*. Chicken studies used saline, dimethyl sulfoxide (DMSO), or sunflower oil as vehicle controls, and some studies included an uninjected control (Table 4). The zebrafish study used water as a vehicle control, the fruit fly study used untreated food as a vehicle control, and the salmon study used water with carrier solvent (methanol) as a vehicle control. Besides PFOA exposure, all control groups were treated similarly to dose groups for each data set.

*Nonmammalian outcomes*. Relevant outcome measures varied across nonmammalian studies and included length, weight, and larval volume (calculated from measurements of length and diameter) (Table 4). Because embryos were directly exposed to PFOA in the nonmammalian model systems, the embryo was used as the unit of statistical analysis, and the total number of embryos analyzed across studies varied between 37 and 378.

The time points of outcome measurement varied: from shortly before time of hatching, shortly after hatching, and multiple time points during larval development.

PFOA exposure delayed hatching and larval emergence in the zebrafish and fruit fly studies and induced mortality in the zebrafish study and in one chicken study. Pipping success (i.e., when a chick breaks its shell) and the developmental stage at embryo death were unaffected by PFOA exposure in one chicken study, whereas in a second chicken study, embryonic mortality was increased but hatchling mortality and hatching success were not affected. The salmon study did not provide details on larval survival rates.

*Nonmammalian risk of bias assessment*. On the basis of our risk of bias assessment, we found that the majority of studies had probably high risk of bias for sequence generation, allocation concealment, and blinding, and probably low risk of bias for selective reporting. Ratings for incomplete outcome data were mixed across studies, and ranged from low to high risk of bias. Finally, all studies had probably low or low risk of bias for conflict of interest and low risk of bias for the “other bias” domain (Figure 3A,B). See Supplemental Material, Tables S40–S45, for details of the risk of bias results for each nonmammalian study.

### Impact of PFOA on Fetal Growth

**Figure 4:**
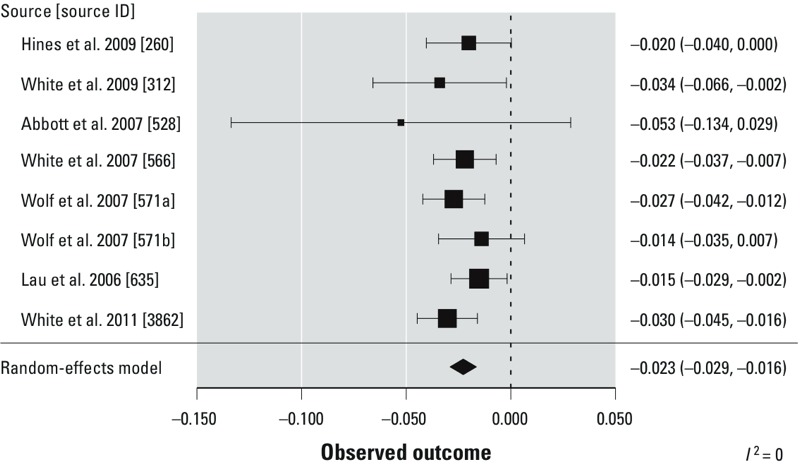
Meta-analysis results from a two-step mixed-effects model using combined relevant mouse studies in which dams were treated with PFOA via gavage and progeny weight was measured at or soon after birth. Data are presented as the mean (95% CI) change in body weight (g) per 1-unit increase in dose (mg/kg BW/day). Each box represents the dose–response slope estimate for a study; the mid­point of the box is the slope estimated for that study, and the box area is proportional to the weight given to each study in the meta-analysis. The diamond is centered at the overall meta-analysis slope estimate. Wolf (2007) was split into two data sets: *a*) cross-foster (exposure on GDs 1–17), and *b*) windows of sensitivity (exposure groups for GDs 7–17, GDs 10–17, GDs 13–17, and GDs 15–17).

*Analysis.* Across the eight data sets determined to be combinable in the meta-analysis, gavage exposure of pregnant mice to increasing concentrations of PFOA was associated with a decrease in birth weight. The combined estimate from the meta-analysis was a change in mean pup birth weight of –0.023 g (95% CI: –0.029, –0.016) per 1-unit increase in dose (mg/kg BW/day) (Figure 4). The *I*^2^ test statistic was calculated to be 0%, indicating no observed heterogeneity between studies that could not be explained by chance; this conclusion was further supported by the *Q* statistic, which produced a nonsignificant *p*-value of 0.73.

From the sensitivity analysis, where we removed one data set at a time, we found relatively small changes in the effect estimate, with a maximum of 9% change in the meta-analysis estimate (from –0.023 to –0.021) when the data set from White et al. (2011) was removed (data not shown). Figure 4 shows that the study of White et al. (2011) resulted in the largest estimate of decreased birth weight among those studies weighted more heavily in the meta-analysis (indicated by the larger size of the mean symbol), so it is not surprising that the removal of this study would have the largest effect on the meta-analysis estimate, and in particular shifting it to a smaller estimate of decreased birth weight. Although the data set of Abbott et al. (2007) had the largest effect estimate, removing that data set had little effect on the meta-analysis because of its small weight. The sensitivity analysis further demonstrated that the 95% CIs were also minimally affected and consistently did not include zero.

**Figure 5:**
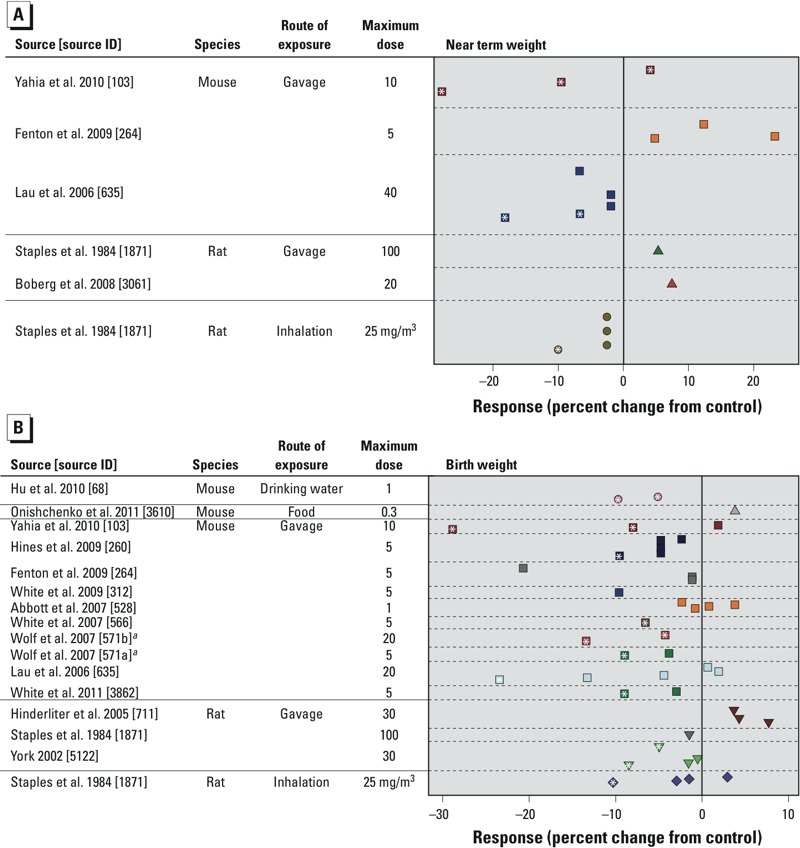
Combined scatter plots of response for each tested dose of PFOA for all included mammalian studies. Response was measured as the percentage of weight change for progeny (*A*) near-term or (*B*) at birth. Each color represents a different study (separated by dashed lines), and each symbol represents a different species or exposure route category. Multiple symbols of the same color represent responses at multiple tested doses within the same study. Doses are given in mg/kg BW/day unless otherwise specified. For each study, doses decrease as the *y*-axis increases and are scaled appropriately (i.e., larger vertical gaps indicate larger gaps between doses); the minimum dose for all studies is zero. See Supplemental Material, Tables S46 and S47, for the 95% CIs for the point estimates shown in the figure. ***^a^***Study split into two data sets: *a*) cross-foster (exposure on GDs 1–17), and *b*) windows of sensitivity (exposure groups for GDs 7–17, GDs 10–17, GDs 13–17, and GDs 15–17). *(Within symbols), *p* < 0.05 compared with the control group.

We created separate scatter plots to summarize all the mammalian study data for near-term, fetal weight measurements (Figure 5A) and for birth weight measurements (Figure 5B). The dose–response data for the nine studies not included in the meta-analysis showed mixed results, generally with lower doses showing increased weight compared with the control group (mostly nonsignificant) and higher doses showing decreased weight (some statistically significant and others not significant) (Figure 5B). The 95% CIs for the mean difference comparing birth weight in the treatment versus control group for each study are presented in Supplemental Material, Tables S46 and S47.

**Figure 6:**
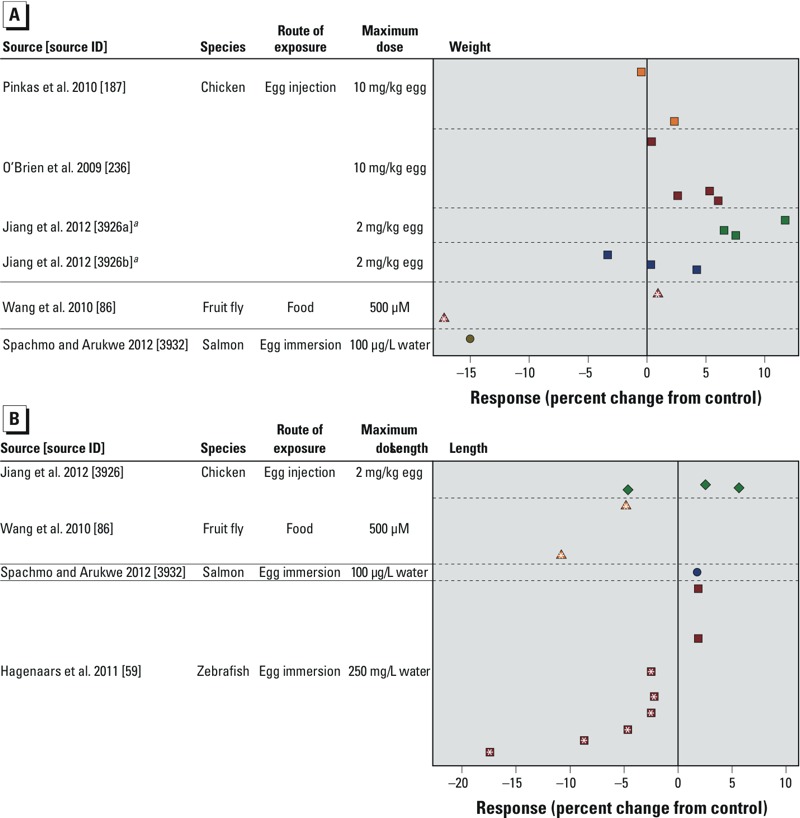
Combined scatter plots of response for each tested dose of PFOA for all included non­mammalian studies. Response was measured as the percentage of (*A*) weight change and (*B*) length change. Each color represents a different study (separated by dashed lines), and each symbol represents a different species or exposure route category. Multiple symbols of the same color represent responses at multiple tested doses within the same study. For each study, doses decrease as the *y*-axis increases and are scaled appropriately (i.e., larger vertical gaps indicate larger gaps between doses); the minimum dose for all studies is zero. See Supplemental Material, Tables S48 and S49, for the 95% CIs for the point estimates shown in the figure. ***^a^***Study split into two data sets based on time of outcome measurement: *a*) embryonic day 19, and *b*) 16–24 hr posthatching. *(Within symbols), *p* < 0.05 compared with the control group.

We also created scatter plots to summarize nonmammalian study data separately for weight measurements (Figure 6A) and for length measurements (Figure 6B). A qualitative evaluation of dose–response data showed mostly nonstatistically significant increases in body weight, even at the highest tested doses. The length data show mixed results, with two studies demonstrating statistically significant decreases in length and the other two studies showing nonsignificant increases in length. The 95% CIs for the mean difference comparing birth weight in the treatment versus control group for each study are presented in Supplemental Material, Tables S48 and S49.

*Quality of evidence.* We downgraded the overall quality rating of the mammalian evidence from “high” to “moderate” based on risk of bias across studies, because the majority of studies were deemed to have “probably high” risk of bias for allocation concealment and blinding. Our ratings and rationales for the overall quality of mammalian evidence are presented in Table 5.

**Table 5:** Mammalian summary of findings, quality of evidence, and strength of evidence.

Factor	Rating	Basis
Risk of bias across studies	–1	“Allocation concealment” and “blinding” risks of bias were *a*) truly present, and *b*) these risks of bias are shown empirically to influence study outcome in preclinical experimental animal studies.
Indirectness	0	Mammalian data are empirically recognized as direct evidence of human health (Kimmel et al. 1984; U.S. EPA 1996) and there are no data to counteract this assumption.
Inconsistency	0	Point estimates across similar studies (e.g., mouse gavage) are consistent with overlapping confidence bounds. Estimates of change in birth weight from studies in the meta-analysis are consistently in the same direction and have low heterogeneity. Results are also consistent in magnitude and direction of effect estimates. Results of the meta-analysis do not appear to be strongly influenced by an individual study.
Imprecision	0	Mammalian data included in the meta-analysis showed relatively small CIs in final estimates. Although some studies did not report CIs, data show statistically significant responses at high doses—indicating small CIs.
Publication bias	0	We found no reason to suspect publication bias. The studies were consistent among their findings regardless of size and funding source; the search was comprehensive, and no unpublished studies were found that presented results out of the range of estimates reported by published studies.
Overall quality of evidence (initial rating is “high”)	Moderate	“High” + (–1) = “moderate”
Summary of findings from meta-analysis	NA	Average change in birth weight = –0.023 g [–0.029, –0.016] per 1-unit increase in dose (mg/kg BW/day)
Summary of findings from qualitative analysis	NA	The dose–response data showed mixed results, generally with lower doses showing increased weight compared with the control group (mostly nonsignificant) and higher doses showing decreased weight (some statistically significant and other not significant).
Overall strength of evidence	Sufficient	
NA, not applicable. Ratings: –1, 1 level downgrade in quality. 0, no change in quality. Studies included in meta-analysis [source ID]: Abbott et al. 2007 [528], Hines et al. 2009 [260], Lau et al. 2006 [635] (birth weight data), White et al. 2007 [566], White et al. 2009 [312], White et al. 2011 [3862], and Wolf et al. 2007 [571] (cross-foster and windows of sensitivity data). Other studies [source ID]: Boberg 2008 et al. [3061], Fenton et al. 2009 [264], Hinderliter et al. 2005 [711], Hu et al. 2010 [68], Lau et al. 2006 [635] (fetal weight data), Onishchenko et al. 2011 [3610], Staples 1984 et al. [1871], Yahia et al. 2010 [103], and York 2002 [5122].		

We downgraded the overall quality rating of the nonmammalian evidence from “high” to “low” because of *a*) risk of bias across studies, given that most studies were deemed to have “probably high” risk of bias for the sequence generation, allocation concealment, and blinding domains; and *b*) indirectness—for the purposes of this case study, we did not have a rationale or evidence to support that all the nonmammalian species and their corresponding routes of exposure were directly applicable model systems for evaluating human fetal growth. Our ratings and rationales for the overall quality of nonmammalian evidence are presented in Table 6.

**Table 6:** Non­mammalian summary of findings, quality of evidence, and strength of evidence.

Factor	Rating	Basis
Risk of bias across studies	–1	“Sequence generation,” “allocation concealment,” and “blinding” risks of bias were: *a*) truly present; and *b*) these risks of bias are shown to empirically influence study outcome in preclinical experimental animal studies.
Indirectness	–1	We lacked an empirical basis supporting that these non­mammalian data were directly relevant to the human health outcome of interest, and the routes of exposure varied from how humans would be exposed to PFOA. Some evidence supports indirectness, in particular: Embryonic development in mammalian organisms (i.e., *in utero* development and live birth) is fundamentally different from development in non­mammalian organisms (i.e., development in egg and hatching), and the route of exposures for the non­mammalian organisms (i.e., eggs injected with or immersed in PFOA-containing solution) are not applicable to humans or other mammalian organisms.
Inconsistency	0	Results appear to divide based on measurement of outcome (weight vs. length); however, results are consistent between comparable studies (comparable for outcome, species, and exposure route).
Imprecision	0	The zebrafish and fruit fly data have a relatively large sample size, and while no confidence bounds are given, the effect estimates are reasonably close to each other (–5% to –20% change). Although some studies did not report CIs, data show statistically significant responses at high doses—indicating small CIs.
Publication bias	0	We found no reason to suspect publication bias. The search was comprehensive, the studies were of various sizes and had various funding sources, and no unpublished studies were found that presented results out of the range of estimates reported by published studies.
Overall quality of evidence (initial rating is “high”)	Low	“High” + (–2) = “Low”
Summary of findings from qualitative analysis	NA	Dose–response data show mostly nonstatistically significant increases in body weight, even at the highest tested doses. The length data show mixed results, with two studies demonstrating statistically significant decreases in length and the other two studies showing statistically nonsignificant increases in length.
NA, not applicable. Ratings: –1, 1 level downgrade in quality. 0, no change in quality. Studies [source ID]: Hagenaars et al. 2011 [59], Jiang et al. 2012 [3926], O’Brien et al. 2009 [236], Pinkas et al. 2010 [187], Spachmo and Arukwe 2012 [3932], and Wang et al. 2010 [86].		

*Strength of evidence rating.* We excluded the nonmammalian data from the final strength of evidence rating. Our rationale was that the nonmammalian evidence was judged to be of low quality for the purposes of addressing our study question, and we had higher quality direct evidence on which to base a decision. Our strength of the evidence considerations were as follows:

Quality of body of evidence: moderate
Direction of effect estimates: decreasing birth weight with increasing exposure to PFOA
Confidence in effect estimates: confidence based on the consistency of the results and overlapping CIs
Other compelling attributes of the data that may influence certainty: none.

We compared these considerations with the definitions in Table 2 and concluded that the animal evidence is sufficient to conclude that exposure to PFOA or its salts adversely affect fetal growth in animals.

## Discussion

### Animal Evidence for PFOA and Fetal Growth

Based on this first application of the Navigation Guide systematic review methodology, we found “sufficient” evidence that fetal developmental exposure to PFOA or its salts reduces fetal growth in animals. Our finding that the data were “sufficient” was based on “moderate” quality mammalian evidence, reduction in mean offspring birth weight from dams exposed to increasing concentrations of PFOA during pregnancy, and our confidence in the effect based on the consistency of the results and overlapping CIs. Analysis of the scatter plots of the studies excluded from the meta-analysis supported that the majority of these studies also found consistently small reductions in measures of fetal growth following maternal exposure to PFOA.

From the meta-analysis of eight mouse gavage data sets, we estimated that exposure of pregnant mice to increasing concentrations of PFOA was associated with a change in mean pup birth weight of –0.023 g (95% CI: –0.029, –0.016) per 1-unit increase in dose (mg/kg BW/day). To assess the biological significance of this estimate, we pooled birth weight measurements from each of the eight control groups to estimate an overall mean birth weight of 1.57 g for the pups in control groups. A 0.023 g decrease in body weight is equivalent to an approximate 1.46% decrease in average body weight per 1-unit increase in PFOA dose. Thus, for example, according to this model, a dose of 10 mg/kg BW/day PFOA to pregnant dams is estimated to result in approximately a 15% decrease in the litter’s average birth weight.

To address the heterogeneity of the available evidence, we limited the meta-analysis to data from mouse studies. The rationale for this decision was based in part on findings from pharmacokinetic studies documenting that the rate of elimination for PFOA is much faster for female rats compared with other mammalian species, including humans (Lau et al. 2007). Many of the studies included in our meta-analysis cited rate of elimination differences as a supporting reason for using mouse model systems. However, responses between mouse model systems may differ as well; evidence suggests that responses to PFOA may vary based on the mouse strain tested. One study noted that the 129S1/SvlmJ strain was more sensitive to PFOA exposure compared with the CD-1 strain (Abbott et al. 2007). We included data from the 129S1/SvlmJ strain in our meta-analysis because, in the absence of evidence supporting which mouse strain best matches human sensitivity to PFOA, there was no evidence to support a premise that humans are less sensitive than the most sensitive mouse. This is further supported by studies of agents known to cause reproductive toxicity, for which “humans appear to be as or more sensitive than the most sensitive animal species tested” (U.S. EPA 1996). Additionally, our sensitivity analysis found removing this study from the meta-analysis resulted in minimal changes in the meta-analysis estimate (< 2%) (data not shown).

The heterogeneity of the nonmammalian animal data precluded combining these studies quantitatively. Our identification of studies among such diverse species was unexpected, and for this case study, we combined all nonmammalian species into a single body of evidence. This did not impede decision making about toxicity of PFOA and fetal growth because more direct mammalian and human data were available. However, for other chemicals, heterogeneous indirect evidence may be the only data available on which to base a decision. This points to the need to anticipate and plan for the analysis of heterogeneous data—including whether it is appropriate to evaluate each species separately—and to determine relevance to human health beforehand in future protocols.

### Application of the Navigation Guide Systematic Review Methodology

We found the application of the Navigation Guide method to be effective in producing a concise statement of health hazard in a systematic and transparent manner. Although our review did not identify any studies relevant to our study question that were published in languages other than English, it is difficult to predict in which cases excluding non-English studies may bias a systematic review (Sterne et al. 2011b). Therefore, for future reviews we would retain this strategy. Moreover, our systematic search identified > 1,900 studies that we did not find in a previous search that we conducted at the initiation of the project using traditional nonsystematic methods, and our improved search strategy nearly doubled the number of studies that met our prespecified inclusion criteria.

Despite a steep learning curve, designing and completing the search, eliminating duplicate records, screening studies, and extracting study characteristics and data took 2–3 months, including time to train review authors. Contact with study authors to obtain additional information took place over the course of approximately 3 months. Risk of bias assessment, data analysis, and evaluation of quality and strength of evidence took approximately 2–3 additional months.

An inevitable limitation of this first case study was that we were simultaneously developing and applying the method. As a result, we did not anticipate or define beforehand all the benchmarks we ultimately used for making judgments when rating the quality and strength of the evidence, and we found that our decision making was more difficult in the absence of prespecified definitions. To guide our judgments when assessing quality and strength of evidence factors that had not been prespecified, we *a*) sought an empirical basis for a judgment; *b*) conducted further analysis (i.e., sensitivity); *c*) relied on GRADE’s principle to be conservative in the judgment of rating down; and *d*) always documented the rationale for our judgment. Anticipating and defining prespecified criteria for as many judgments as possible will improve the method; however, it seems unlikely that all judgments can be anticipated. Thus, the principles we used for judgments subsequent to the analysis will be integrated into future protocols to transparently allow for such circumstances.

### Challenges in Translating Experimental Animal Evidence into Improved Health Outcomes

In applying the Navigation Guide systematic review methodology, we found that the high prevalence of suboptimal study design and reporting in experimental animal studies that has been empirically documented in the preclinical literature (Bebarta et al. 2003; Landis et al. 2012; Macleod et al. 2004, 2008; McPartland et al. 2007; van der Worp and Macleod 2011; van der Worp et al. 2007; Vesterinen et al. 2011) may also be prevalent in the experimental animal data that inform decision making in environmental health. In nearly all of the studies included in our review, direct evidence to support risk of bias ratings, such as clear descriptions of randomization or blinding methods, was missing. Furthermore, many studies failed to report some of the basic data necessary for interpretation of results and incorporation into meta-analysis. For example, multiple studies failed to report data such as the number of animals included in outcome measurements (e.g., number of litters assessed, number of pups per litter), details on how offspring were weighed (e.g., individually, as a whole litter), or the time point of outcome assessment (e.g., clear definition of PND1, monitoring of parturition). To create scatter plots and perform a meta-analysis, we needed to contact the lead author of every study to obtain missing data. Fortunately, authors for most of the studies responded, and many generously took the time and effort to provide raw data for inclusion in this review. Our follow-up with the authors indicated that many of these missing data were a result of deficiencies in reporting and point to the need to include contacting study authors as a step in the protocol.

These findings underscore the urgency of calls for improved experimental-animal study design and reporting in the preclinical arena (Beronius et al. 2014; Krauth et al. 2013; Landis et al. 2012; van der Worp and Macleod 2011; Vesterinen et al. 2010, 2011). To this end, a major stakeholder meeting by the National Institute of Neurological Disorders and Stroke found that at a minimum, studies should report on sample-size estimation, whether and how animals were randomized, whether investigators were blind to the treatment, and the handling of data (Landis et al. 2012). It will be important for environmental health scientists and journals that publish environmental health research to help support these nascent efforts to advance the translational relevance of animal evidence into improved health outcomes (Howells and Macleod 2013; Macleod et al. 2009; van der Worp et al. 2010; Vesterinen et al. 2011).

## Summary and Conclusion

This case study documents that the Navigation Guide methodology can be used to effectively apply the rigor of evidence synthesis methods in use by the clinical sciences to questions in environmental health. The Navigation Guide methodology does not eliminate the need for expert judgment, but it does *a*) make clear the evidence that informs the authors’ judgments and *b*) require transparency and an explicit accounting of the judgments involved.

In addition to this review of the animal evidence, a separate systematic review was conducted evaluating the human evidence relevant to PFOA exposure and fetal growth, which resulted in a “sufficient” evidence of toxicity rating (Johnson et al. 2014). In another paper, the strength of the evidence ratings from the nonhuman and human evidence were combined according to the factors specified in the Navigation Guide (Woodruff et al. 2011a), resulting in an overall conclusion by the review authors that human exposure to PFOA is “known to be toxic” to human reproduction and development based on “sufficient” evidence of decreased fetal growth in both human and nonhuman mammalian species (Lam et al. 2014). Together, these reviews demonstrate the utility of the Navigation Guide in systematically approaching a complex body of scientific evidence.

The ultimate goal of our efforts is to refine the Navigation Guide systematic review methodology across diverse streams of evidence and to support the development of recommendations for prevention in clinical and policy spheres. As has been demonstrated in the clinical field, the adoption of systematic and transparent methods to synthesize the scientific evidence in the environmental health field would speed incorporation of research into decision making.

## Supplemental Material

(581 KB) PDF
