# Supplementary material for: The Navigation Guide—Evidence-Based Medicine Meets Environmental Health: Systematic Review of Nonhuman Evidence for PFOA Effects on Fetal Growth
Source: Environ Health Perspect. 2014 Jun 25;122(10):1015–27. doi: 10.1289/ehp.1307177 (PMC4181920; doi:10.1289/ehp.1307177)
Supplement: (581 KB) PDF [file ehp.1307177.s001.508.pdf]

Environmental Health Perspectives doi:10.1289/ehp.1307177

Supplemental Material

The Navigation Guide—Evidence-Based Medicine Meets Environmental Health: Systematic Review of Nonhuman Evidence for PFOA Effects on Fetal Growth

Erica Koustas, Juleen Lam, Patrice Sutton, Paula I. Johnson, Dylan S. Atchley, Saunak Sen,

Karen A. Robinson, Daniel A. Axelrad, and Tracey J. Woodruff

Table of Contents **Page**

Table S1: PubMed search terms. **4**

Table S2: Web of Science search terms. **7**

Table S3: Search strategy. **9**

Table S4: Characteristics of Hu et al. 2010 (study ID 68). **10**

Table S5: Characteristics of Yahia et al. 2010 (study ID 103). **11**

Table S6: Characteristics of Hines et al. 2009 (study ID 260). **12**

Table S7: Characteristics of Fenton et al. 2009 (study ID 264). **13**

Table S8: Characteristics of White et al. 2009 (study ID 312). **14**

Table S9: Characteristics of Abbott et al. 2007 (study ID 528). **15**

Table S10: Characteristics of White et al. 2007 (study ID 566). **16**

Table S11: Characteristics of Wolf et al. 2007 (study ID 571). **17**

Table S12: Characteristics of Lau et al. 2006 (study ID 635). **18**

Table S13: Characteristics of Hinderliter et al. 2005 (study ID 711). **19**

Table S14: Characteristics of Staples et al. 1984 (study ID 1871). **20**

Table S15: Characteristics of Boberg et al. 2008 (study ID 3061). **21**

Table S16: Characteristics of Onishchenko et al. 2011 (study ID 3610). **22**

Table S17: Characteristics of White et al. 2011 (study ID 3862). **23**

Table S18: Characteristics of York 2002 (study ID 5122). **24**

Table S19: Characteristics of Hagenaars et al. 2011 (study ID 59). **25**

Table S20: Characteristics of Wang et al. 2010 (study ID 86). **26**

Table S21: Characteristics of Pinkas et al. 2010 (study ID 187). **27**

Table S22: Characteristics of O'Brien et al. 2009 (study ID 236). **28**

Table S23: Characteristics of Jiang et al. 2012 (study ID 3926). **29**

Table S24: Characteristics of Spachmo and Arukwe 2012 (study ID 3932). **30**

Table S25: Risk of bias summary of Hu et al. 2010 (study ID 68). **31**

Table S26: Risk of bias summary of Yahia et al. 2010 (study ID 103). **32**

Table S27: Risk of bias summary of Hines et al. 2009 (study ID 260). **33**

Table S28: Risk of bias summary of Fenton et al. 2009 (study ID 264). **34**

Table S29: Risk of bias summary of White et al. 2009 (study ID 312). **35**

Table S30: Risk of bias summary of Abbott et al. 2007 (study ID 528). **36**

Table S31: Risk of bias summary of White et al. 2007 (study ID 566). **37**

Table S32: Risk of bias summary of Wolf et al. 2007 (study ID 571). **38**

Table S33: Risk of bias summary of Lau et al. 2006 (study ID 635). **39**

Table S34: Risk of bias summary of Hinderliter et al. 2005 (study ID 711). **40**

Table S35: Risk of bias summary of Staples et al. 1984 (study ID 1871). **41**

Table S36: Risk of bias summary of Boberg et al. 2008 (study ID 3061). **42**

Table S37: Risk of bias summary of Onishchenko et al. 2011 (study ID 3610). **43**

Table S38: Risk of bias summary of White et al. 2011 (study ID 3862). **44**

Table S39: Risk of bias summary of York 2002 (study ID 5122). **45**

Table S40: Risk of bias summary of Hagenaars et al. 2011 (study ID 59). **46**

Table S41: Risk of bias summary of Wang et al. 2010 (study ID 86). **47**

Table S42: Risk of bias summary of Pinkas et al. 2010 (study ID 187). **48**

Table S43: Risk of bias summary of O'Brien et al. 2009 (study ID 236). **49**

Table S44: Risk of bias summary of Jiang et al. 2012 (study ID 3926). **50**

Table S45: Risk of bias summary of Spachmo and Arukwe 2012 (study ID 3932). **51**

Table S46: 95% Confidence interval estimates for mammalian fetal weight measurements, for each tested dose of PFOA (see Figure 5A). **52**

Table S47: 95% Confidence interval estimates for mammalian birth weight measurements, for each tested dose of PFOA (see Figure 5B). **53**

Table S48: 95% Confidence interval estimates for non-mammalian weight measurements, for each tested dose of PFOA (see Figure 6A). **55**

Table S49: 95% Confidence interval estimates for non-mammalian length measurements, for each tested dose of PFOA (see Figure 6B). **56**

Instructions for making risk of bias determinations **57**

1. Sequence generation *57*

2. Allocation concealment *58*

3. Blinding of personnel and outcome assessors *59*

4. Incomplete outcome data *60*

5. Selective outcome reporting *61*

6. Other potential threats to validity *62*

7. Conflict of interest *63*

References **66**

Table S1: PubMed search terms.

Search	PubMed
#1<br><br>Substance terms	335-67-1 [rn] OR perfluorooctanoic acid [nm] OR (perfluorooctanoic acid [tiab] OR perfluorooctanoic acids [tiab]) OR (perfluoroctanoic acid [tiab] OR perfluoroctanoic acids [tiab]) OR (perfluoro-n-octanoic acid [tiab] OR perfluoro-n-octanoic acids [tiab]) OR (pentadecafluorooctanoic acid [tiab] OR pentadecafluorooctanoic acids [tiab]) OR APFO [tiab] OR (perfluorinated [tiab] AND octanoic acid [tiab]) OR (perfluorinated [tiab] AND octanoic acids [tiab]) OR (perfluorooctanoate [tiab] OR perfluorooctanoates [tiab]) OR perfluorooctanoyl chloride [tiab] OR PFOA [tiab] OR (fluorinated telomer alcohol [tiab] OR fluorinated telomer alcohols [tiab]) OR (fluoro-telomer alcohol [tiab] OR fluoro-telomer alcohols [tiab]) OR (fluorocarbon emulsion [tiab] OR fluorocarbon emulsions [tiab]) OR (perfluorocarbon [tiab] OR perfluorocarbons [tiab]) OR (fluorocarbon polymer [tiab] OR fluorocarbon polymers [tiab]) OR (fluorinated polymer [tiab] OR fluorinated polymers [tiab]) OR octanoic acids [mh] OR (octanoic acid [tiab] OR octanoic acids [tiab]) OR caprylates [mh] OR (caprylate [tiab] OR caprylates [tiab]) OR (polyfluoroalkyl [tiab] OR polyfluoroalkyls [tiab] OR polyfluoroalkylated [tiab]) OR PFAA [tiab] OR (perfluoroalkyl chemical [tiab] OR perfluoroalkyl chemicals [tiab]) OR (c8 [tiab] AND perfluorinated [tiab]) OR (fluoropolymer [tiab] OR fluoropolymers [tiab] OR fluoropolymeric [tiab]) OR (fluorosurfactant [tiab] OR fluorosurfactants [tiab]) OR (perfluorochemical [tiab] OR perfluorochemicals [tiab]) OR PFCs [tiab] OR (perfluoroalkyl carboxylate [tiab] OR perfluoroalkyl carboxylates [tiab]) OR (perfluorocarboxylate [tiab] OR perfluorocarboxylates [tiab]) OR PFCA [tiab] OR (perfluorinated carboxylic acid [tiab] OR perfluorinated carboxylic acids [tiab]) OR FC 143 [tiab] OR (pentadecafluorooctanoate [tiab] OR pentadecafluorooctanoates [tiab])
#2<br><br>Experimental animal terms (modified from Hooijmans et al. 2010)	("animal experimentation"[MeSH Terms] OR "models, animal"[MeSH Terms] OR "invertebrates"[MeSH Terms] OR "Animals"[Mesh:noexp] OR "animal population groups"[MeSH Terms] OR "chordata"[MeSH Terms:noexp] OR "chordata, nonvertebrate"[MeSH Terms] OR "vertebrates"[MeSH Terms:noexp] OR "amphibians"[MeSH Terms] OR "birds"[MeSH Terms] OR "fishes"[MeSH Terms] OR "reptiles"[MeSH Terms] OR "mammals"[MeSH Terms:noexp] OR "primates"[MeSH Terms:noexp] OR "artiodactyla"[MeSH Terms] OR "carnivora"[MeSH Terms] OR "cetacea"[MeSH Terms] OR "chiroptera"[MeSH Terms] OR "elephants"[MeSH Terms] OR "hyraxes"[MeSH Terms] OR "insectivora"[MeSH Terms] OR "lagomorpha"[MeSH Terms] OR "marsupialia"[MeSH Terms] OR "monotremata"[MeSH Terms] OR "perissodactyla"[MeSH Terms] OR "rodentia"[MeSH Terms] OR "scandentia"[MeSH Terms] OR "sirenia"[MeSH Terms] OR "xenarthra"[MeSH Terms] OR "haplorhini"[MeSH Terms:noexp] OR "strepsirhini"[MeSH Terms] OR "platyrrhini"[MeSH Terms] OR "tarsii"[MeSH Terms] OR "catarrhini"[MeSH Terms:noexp] OR "cercopithecidae"[MeSH Terms] OR "hylobatidae"[MeSH Terms] OR "hominidae"[MeSH Terms:noexp] OR "gorilla gorilla"[MeSH Terms] OR "pan paniscus"[MeSH Terms] OR "pan troglodytes"[MeSH Terms] OR "pongo pygmaeus"[MeSH Terms]) OR (animals[tiab] OR animal[tiab] OR mice[Tiab] OR mus[Tiab] OR mouse[Tiab] OR murine[Tiab] OR woodmouse[tiab] OR rats[Tiab] OR rat[Tiab] OR murinae[Tiab] OR muridae[Tiab] OR cottonrat[tiab] OR cottonrats[tiab] OR hamster[tiab] OR hamsters[tiab] OR cricetinae[tiab] OR rodentia[Tiab] OR rodent[Tiab] OR rodents[Tiab] OR pigs[Tiab] OR pig[Tiab] OR swine[tiab] OR swines[tiab] OR piglets[tiab] OR piglet[tiab] OR boar[tiab] OR boars[tiab] OR "sus scrofa"[tiab] OR ferrets[tiab] OR ferret[tiab] OR polecat[tiab] OR polecats[tiab] OR "mustela putorius"[tiab] OR "guinea pigs"[Tiab] OR "guinea pig"[Tiab] OR cavia[Tiab] OR callithrix[Tiab] OR marmoset[Tiab] OR marmosets[Tiab] OR cebuella[Tiab] OR hapale[Tiab] OR octodon[Tiab] OR chinchilla[Tiab] OR chinchillas[Tiab] OR gerbillinae[Tiab] OR gerbil[Tiab] OR gerbils[Tiab] OR jird[Tiab] OR jirds[Tiab] OR merione[Tiab] OR meriones[Tiab] OR

Search	PubMed
	rabbits[Tiab] OR rabbit[Tiab] OR hares[Tiab] OR hare[Tiab] OR diptera[Tiab] OR flies[Tiab] OR fly[Tiab] OR dipteral[Tiab] OR drosphila[Tiab] OR drosophilidae[Tiab] OR cats[Tiab] OR cat[Tiab] OR carus[Tiab] OR felis[Tiab] OR nematoda[Tiab] OR nematode[Tiab] OR nematodes[Tiab] OR sipunculida[Tiab] OR dogs[Tiab] OR dog[Tiab] OR canine[Tiab] OR canines[Tiab] OR canis[Tiab] OR sheep[Tiab] OR sheeps[Tiab] OR mouflon[Tiab] OR mouflons[Tiab] OR ovis[Tiab] OR goats[Tiab] OR goat[Tiab] OR capra[Tiab] OR capras[Tiab] OR rupicapra[Tiab] OR chamois[Tiab] OR haplorhini[Tiab] OR monkey[Tiab] OR monkeys[Tiab] OR anthropoidea[Tiab] OR anthropoids[Tiab] OR saguinus[Tiab] OR tamarin[Tiab] OR tamarins[Tiab] OR leontopithecus[Tiab] OR hominidae[Tiab] OR ape[Tiab] OR apes[Tiab] OR pan[Tiab] OR paniscus[Tiab] OR "pan paniscus"[Tiab] OR bonobo[Tiab] OR bonobos[Tiab] OR troglodytes[Tiab] OR "pan troglodytes"[Tiab] OR gibbon[Tiab] OR gibbons[Tiab] OR siamang[Tiab] OR siamangs[Tiab] OR nomascus[Tiab] OR symphalangus[Tiab] OR chimpanzee[Tiab] OR chimpanzees[Tiab] OR prosimians[Tiab] OR "bush baby"[Tiab] OR prosimian[Tiab] OR bush babies[Tiab] OR galagos[Tiab] OR galago[Tiab] OR pongidae[Tiab] OR gorilla[Tiab] OR gorillas[Tiab] OR pongo[Tiab] OR pygmaeus[Tiab] OR "pongo pygmaeus"[Tiab] OR orangutans[Tiab] OR lemur[Tiab] OR lemurs[Tiab] OR lemuridae[Tiab] OR horse[Tiab] OR horses[Tiab] OR equus[Tiab] OR cow[Tiab] OR calf[Tiab] OR bull[Tiab] OR chicken[Tiab] OR chickens[Tiab] OR gallus[Tiab] OR quail[Tiab] OR bird[Tiab] OR birds[Tiab] OR quails[Tiab] OR poultry[Tiab] OR poultries[Tiab] OR fowl[Tiab] OR fowls[Tiab] OR reptile[Tiab] OR reptilia[Tiab] OR reptiles[Tiab] OR snakes[Tiab] OR snake[Tiab] OR lizard[Tiab] OR lizards[Tiab] OR alligator[Tiab] OR alligators[Tiab] OR crocodile[Tiab] OR crocodiles[Tiab] OR turtle[Tiab] OR turtles[Tiab] OR amphibian[Tiab] OR amphibians[Tiab] OR amphibia[Tiab] OR frog[Tiab] OR frogs[Tiab] OR bombina[Tiab] OR salientia[Tiab] OR toad[Tiab] OR toads[Tiab] OR "epidalea calamita"[Tiab] OR salamander[Tiab] OR salamanders[Tiab] OR eel[Tiab] OR eels[Tiab] OR fish[Tiab] OR fishes[Tiab] OR pisces[Tiab] OR catfish[Tiab] OR catfishes[Tiab] OR siluriformes[Tiab] OR arius[Tiab] OR heteropneustes[Tiab] OR sheatfish[Tiab] OR perch[Tiab] OR perches[Tiab] OR percidae[Tiab] OR perca[Tiab] OR trout[Tiab] OR trouts[Tiab] OR char[Tiab] OR chars[Tiab] OR salvelinus[Tiab] OR "fathead minnow"[Tiab] OR minnow[Tiab] OR cyprinidae[Tiab] OR carps[Tiab] OR carp[Tiab] OR zebrafish[Tiab] OR zebrafishes[Tiab] OR goldfish[Tiab] OR goldfishes[Tiab] OR guppy[Tiab] OR guppies[Tiab] OR chub[Tiab] OR chubs[Tiab] OR tinca[Tiab] OR barbels[Tiab] OR barbus[Tiab] OR pimephales[Tiab] OR promelas[Tiab] OR "poecilia reticulata"[Tiab] OR mullet[Tiab] OR mullets[Tiab] OR seahorse[Tiab] OR seahorses[Tiab] OR mugil curema[Tiab] OR atlantic cod[Tiab] OR shark[Tiab] OR sharks[Tiab] OR catshark[Tiab] OR anguilla[Tiab] OR salmonid[Tiab] OR salmonids[Tiab] OR whitefish[Tiab] OR whitefishes[Tiab] OR salmon[Tiab] OR salmons[Tiab] OR sole[Tiab] OR solea[Tiab] OR "sea lamprey"[Tiab] OR lamprey[Tiab] OR lampreys[Tiab] OR pumpkinseed[Tiab] OR sunfish[Tiab] OR sunfishes[Tiab] OR tilapia[Tiab] OR tilapias[Tiab] OR turbot[Tiab] OR turbots[Tiab] OR flatfish[Tiab] OR flatfishes[Tiab] OR sciuridae[Tiab] OR squirrel[Tiab] OR squirrels[Tiab] OR chipmunk[Tiab] OR chipmunks[Tiab] OR suslik[Tiab] OR susliks[Tiab] OR vole[Tiab] OR voles[Tiab] OR lemming[Tiab] OR lemmings[Tiab] OR muskrat[Tiab] OR muskrats[Tiab] OR lemmus[Tiab] OR otter[Tiab] OR otters[Tiab] OR marten[Tiab] OR martens[Tiab] OR martes[Tiab] OR weasel[Tiab] OR badger[Tiab] OR badgers[Tiab] OR ermine[Tiab] OR mink[Tiab] OR minks[Tiab] OR sable[Tiab] OR sables[Tiab] OR gulo[Tiab] OR gulos[Tiab] OR wolverine[Tiab] OR wolverines[Tiab] OR mustela[Tiab] OR llama[Tiab] OR llamas[Tiab] OR alpaca[Tiab] OR alpacas[Tiab] OR camelid[Tiab] OR camelids[Tiab] OR guanaco[Tiab] OR guanacos[Tiab] OR chiroptera[Tiab] OR chiropteras[Tiab] OR bat[Tiab] OR bats[Tiab] OR fox[Tiab] OR foxes[Tiab] OR iguana[Tiab] OR iguanas[Tiab] OR xenopus laevis[Tiab] OR parakeet[Tiab] OR

Search	PubMed
	parakeets[Tiab] OR parrot[Tiab] OR parrots[Tiab] OR donkey[Tiab] OR donkeys[Tiab] OR mule[Tiab] OR mules[Tiab] OR zebra[Tiab] OR zebras[Tiab] OR shrew[Tiab] OR shrews[Tiab] OR bison[Tiab] OR bisons[Tiab] OR buffalo[Tiab] OR buffaloes[Tiab] OR deer[Tiab] OR deers[Tiab] OR bear[Tiab] OR bears[Tiab] OR panda[Tiab] OR pandas[Tiab] OR "wild hog"[Tiab] OR "wild boar"[Tiab] OR fitchew[Tiab] OR fitch[Tiab] OR beaver[Tiab] OR beavers[Tiab] OR jerboa[Tiab] OR jerboas[Tiab] OR capybara[Tiab] OR capybaras[Tiab])
#3<br><br>Reproductive/developmental toxicity terms	developmental biology [mh] OR developmental biology [tiab] OR embryonic and fetal development [mh] OR (embryonic [tiab] OR embryonically [tiab]) OR fetal development [tiab] OR growth and development [mh] OR growth and development [subheading] OR (development [tiab] OR developmental [tiab] OR developmentally [tiab]) OR embryology [mh] OR embryology [tiab] OR ecotoxicology [mh] OR ecotoxicology [tiab] OR ecology [mh] OR (ecology [tiab] OR ecological [tiab] OR ecologically [tiab]) OR toxicology [mh] OR (toxicology [tiab] OR toxicological [tiab] OR toxicologically [tiab]) OR toxicogenetics [mh] OR (toxicogenetic [tiab] OR toxicogenetics [tiab]) OR growth [mh] OR growth [tiab] OR environmental pollutants [mh] OR (environmental pollutant [tiab] OR environmental pollutants [tiab]) OR body weight [mh] OR (weight [tiab] OR weights [tiab] OR weighed [tiab]) OR embryo loss [mh] OR (embryo loss [tiab] OR embryo losses [tiab]) OR fetal resorption [mh] OR (fetal resorption [tiab] OR fetal resorptions [tiab]) OR gestational age [mh] OR (gestational age [tiab] OR gestational ages [tiab]) OR litter size [mh] OR (litter size [tiab] OR litter sizes [tiab]) OR endocrine disruptors [mh] OR (endocrine disruptor [tiab] OR endocrine disruptors [tiab] OR endocrine disruption [tiab]) OR reproduction [mh] OR reproduction [tiab] OR toxicity [subheading] OR toxicity [tiab] OR (toxic [tiab] OR toxics [tiab])
**#4**	**#1 AND #2 AND #3**

Table S2: Web of Science search terms.

**Search**	**Web of Science**
#1<br><br>Substance terms	TS=((perfluorooctanoic acid OR perfluorooctanoic acids) OR (perfluoroctanoic acid OR perfluoroctanoic acids) OR (perfluoro-n-octanoic acid OR perfluoro-n-octanoic acids) OR (pentadecafluorooctanoic acid OR pentadecafluorooctanoic acids) OR APFO OR (perfluorinated AND octanoic acid) OR (perfluorinated AND octanoic acids) OR (perfluorooctanoate OR perfluorooctanoates) OR perfluorooctanoyl chloride OR PFOA OR (fluorinated telomer alcohol OR fluorinated telomer alcohols) OR (fluoro-telomer alcohol OR fluoro-telomer alcohols) OR (fluorocarbon emulsion OR fluorocarbon emulsions) OR (perfluorocarbon OR perfluorocarbons) OR (fluorocarbon polymer OR fluorocarbon polymers) OR (fluorinated polymer OR fluorinated polymers) OR (octanoic acid OR octanoic acids) OR (caprylate OR caprylates) OR (polyfluoroalkyl OR polyfluoroalkyls OR polyfluoroalkylated) OR PFAA OR (perfluoroalkyl chemical OR perfluoroalkyl chemicals) OR (c8 AND perfluorinated) OR (fluoropolymer OR fluoropolymers OR fluoropolymeric) OR (fluorosurfactant OR fluorosurfactants) OR (perfluorochemical OR perfluorochemicals) OR PFCs OR (perfluoroalkyl carboxylate OR perfluoroalkyl carboxylates) OR (perfluorocarboxylate OR perfluorocarboxylates) OR PFCA OR (perfluorinated carboxylic acid OR perfluorinated carboxylic acids) OR FC 143 OR (pentadecafluorooctanoate OR pentadecafluorooctanoates))
#2<br><br>Experimental animal terms (modified from Hooijmans et al. 2010)	TS=(animals OR animal OR mice OR mus OR mouse OR murine OR woodmouse OR rats OR rat OR murinae OR muridae OR cottonrat OR cottonrats OR hamster OR hamsters OR cricetinae OR rodentia OR rodent OR rodents OR pigs OR pig OR swine OR swines OR piglets OR piglet OR boar OR boars OR "sus scrofa" OR ferrets OR ferret OR polecat OR polecats OR "mustela putorius" OR "guinea pigs" OR "guinea pig" OR cavia OR callithrix OR marmoset OR marmosets OR cebuella OR hapale OR octodon OR chinchilla OR chinchillas OR gerbillinae OR gerbil OR gerbils OR jird OR jirds OR merione OR meriones OR rabbits OR rabbit OR hares OR hare OR diptera OR flies OR fly OR dipteral OR drosphila OR drosophilidae OR cats OR cat OR carus OR felis OR nematoda OR nematode OR nematodes OR sipunculida OR dogs OR dog OR canine OR canines OR canis OR sheep OR sheeps OR mouflon OR mouflons OR ovis OR goats OR goat OR capra OR capras OR rupicapra OR chamois OR haplorhini OR monkey OR monkeys OR anthropoidea OR anthropoids OR saguinus OR tamarin OR tamarins OR leontopithecus OR hominidae OR ape OR apes OR pan OR paniscus OR "pan paniscus" OR bonobo OR bonobos OR troglodytes OR "pan troglodytes" OR gibbon OR gibbons OR siamang OR siamangs OR nomascus OR symphalangus OR chimpanzee OR chimpanzees OR prosimians OR "bush baby" OR prosimian OR bush babies OR galagos OR galago OR pongidae OR gorilla OR gorillas OR pongo OR pygmaeus OR "pongo pygmaeus" OR orangutans OR lemur OR lemurs OR lemuridae OR horse OR horses OR equus OR cow OR calf OR bull OR chicken OR chickens OR gallus OR quail OR bird OR birds OR quails OR poultry OR poultries OR fowl OR fowls OR reptile OR reptilia OR reptiles OR snakes OR snake OR lizard OR lizards OR alligator OR alligators OR crocodile OR crocodiles OR turtle OR turtles OR amphibian OR amphibians OR amphibia OR frog OR frogs OR bombina OR salientia OR toad OR toads OR "epidalea calamita" OR salamander OR salamanders OR eel OR eels OR fish OR fishes OR pisces OR catfish OR catfishes OR siluriformes OR arius OR heteropneustes OR sheatfish OR perch OR perches OR percidae OR perca OR trout OR trouts OR char OR chars OR salvelinus OR "fathead minnow" OR minnow OR cyprinidae OR carps OR carp OR zebrafish OR zebrafishes OR goldfish OR goldfishes OR guppy OR guppies OR chub OR chubs OR tinca OR barbels OR barbus OR pimephales OR promelas OR "poecilia reticulata" OR mullet OR mullets OR seahorse OR seahorses OR mugil curema OR atlantic cod OR shark OR sharks OR catshark OR anguilla OR salmonid OR salmonids OR whitefish OR whitefishes OR salmon OR salmons OR sole OR solea OR "sea lamprey" OR lamprey OR

Search	Web of Science
	lampreys OR pumpkinseed OR sunfish OR sunfishes OR tilapia OR tilapias OR turbot OR turbots OR flatfish OR flatfishes OR sciuridae OR squirrel OR squirrels OR chipmunk OR chipmunks OR suslik OR susliks OR vole OR voles OR lemming OR lemmings OR muskrat OR muskrats OR lemmus OR otter OR otters OR marten OR martens OR martes OR weasel OR badger OR badgers OR ermine OR mink OR minks OR sable OR sables OR gulo OR gulos OR wolverine OR wolverines OR mustela OR llama OR llamas OR alpaca OR alpacas OR camelid OR camelids OR guanaco OR guanacos OR chiroptera OR chiropteras OR bat OR bats OR fox OR foxes OR iguana OR iguanas OR xenopus laevis OR parakeet OR parakeets OR parrot OR parrots OR donkey OR donkeys OR mule OR mules OR zebra OR zebras OR shrew OR shrews OR bison OR bisons OR buffalo OR buffaloes OR deer OR deers OR bear OR bears OR panda OR pandas OR "wild hog" OR "wild boar" OR fitchew OR fitch OR beaver OR beavers OR jerboa OR jerboas OR capybara OR capybaras)
#3<br><br>Reproductive/developmental toxicity terms	TS=(developmental biology OR (embryonic OR embryonically) OR (development OR developmental OR developmentally) OR embryology OR ecotoxicology OR (ecology OR ecological OR ecologically) OR (toxicology OR toxicological OR toxicologically) OR (toxicogenetic OR toxicogenetics) OR growth OR (environmental pollutant OR environmental pollutants) OR (weight OR weights OR weighed) OR (embryo loss OR embryo losses) OR (fetal resorption OR fetal resorptions) OR (gestational age OR gestational ages) OR (litter size OR litter sizes) OR (endocrine disruptor OR endocrine disruptors OR endocrine disruption) OR reproduction OR (toxicity OR toxic OR toxics))
**#4**	**#1 AND #2 AND #3**

Table S3: Search strategy

**Source**[a]	**Hits**[b]
PubMed	1462
Web of Science (Thompson Reuters)	1060
Agency for Toxic Substances and Disease Registry (ATSDR) Interaction Profiles and Toxicological Profiles	1
Developmental and Reproductive Toxicology Database (DART)	10
EPA Science Inventory	88
USEPA Health and Environmental Studies Online (HERO)	52
National Institute for Occupational Safety and Health publications database (NIOSHTIC-2)	7
Toxicology Literature Online (TOXLINE)	85
Toxic Substances Control Act Test Submissions (TSCATS)	2

[a]Table presents sources for which search results were returned; sources that did not return search results follow: CalEPA Office of Environmental Health Hazard Assessment Risk Assessment; Chem IDplus Advanced; Chemspider; Chemical Carcinogenesis Research Information System (CCRIS); EPA Acute Exposure Guideline Levels Chemicals; EPA Integrated Risk Information System (IRIS); EPA National Environmental Publications Internet Site (NEPIS); EPA National Service Center for Environmental Publications (NSCEP); EPA Substance Registry Services; Environmental Mutagen Information Center (EMIC); European Chemicals Agency; Genetic Toxicology Data Bank (GENE-TOX); Health Canada First Priority Substances List (PSL1) Assessments; Health Canada Second Priority Substances List (PSL2) Assessments; Hazardous Substances Data Bank (HSDB); IARC Monographs on the Evaluation of Carcinogenic Risks to Humans; International Life Sciences Institute (ILSI); International Programme on Chemical Safety (IPCS); International Toxicity Estimates for Risk (ITER); US National Toxicology Program Management Status Report; US National Toxicology Program Results and Status Search; US National Toxicology Program Report on Carcinogens; Toxicology Excellence for Risk Assessment; Toxicology Data Network (TOXNET); NIOSH Registry of Toxic Effects of Chemical Substances (RTECS);WHO Concise International Chemical Assessment Documents; WHO Environmental Health Criteria. For additional information, see review protocol. [b]PubMed and Web of Science searches were performed on February 3, 2012; all remaining database searches were performed January 23, 2012-February 6, 2012

Table S4: Characteristics of Hu et al. 2010 (study ID 68).

**Study Element**	**Description**
Methods	Mouse developmental toxicological and immunotoxicological study
Participants	Wild-type C57Bl/6 mice<br>Timed-pregnant GD6 animals obtained from supplier<br>Total number of dams allocated: 48
Exposure	Dams treated with PFOA, ammonium salt (CAS# 3825-26-1) in drinking water (a*d libitum* access) from GD 6 to GD 17.<br>Exposure groups:<br>• 2 dose groups = 0.05, 1 mg PFOA/kg body weight/day; 16 dams each<br>• 1 control group = non-treated water; 16 dams
Outcomes	1. Birth weight (g) - individual pups weighed at PND2 (birth occurred previous day or night).<br>Number of dams analyzed:<br>• 10 for each exposure group<br>Not included in review: dam and offspring weight gain; organ weights; IgM and IgG antibody titers; serum PFOA concentrations
Notes	Author responded to requests for additional information. Raw data provided by study author.<br>Litter sizes were statistically equal across doses and control groups.

Table S5: Characteristics of Yahia et al. 2010 (study ID 103).

Study Element	Description
Methods	Mouse developmental and reproductive toxicological study
Participants	Wild-type ICR (CD-1) mice<br>In-house breeding protocol<br>Total number of dams allocated: Unclear
Exposure	Experimental groups:<br>• Prenatal time point: dams treated with PFOA via daily gavage from GD0 to GD17.<br>• Postnatal time point: dams treated with PFOA via daily gavage from GD0 to GD18.<br>Exposure groups:<br>• 3 dose groups = 1, 5, 10 mg PFOA/kg body weight/day<br>• 1 control group = deionized water
Outcomes	1. Fetal weight (g) – individual fetuses weighed at GD18.<br>Number of dams analyzed:<br>• 7, 9, 5, 8 for control, 1, 5, 10 mg/kg PFOA groups, respectively<br>2. Neonatal weight (g) – unclear at what time point pups were weighed.<br>Number of dams analyzed:<br>• 5 for each exposure group<br>Not included in review: maternal organ effects; effects on maternal serum biochemical parameters; maternal weight; survival of offspring
Notes	Authors did not respond to requests for additional information. Failure to report pertinent study details resulted in exclusion from quantitative analysis for birth weight outcome, despite meeting inclusion criteria otherwise. Authors did not specify chemical form of PFOA (assume CAS# 335-67-1). For prenatal time point, no significant effects on survival. For postnatal time point, delayed delivery, high rate of stillborn birth (58%), and remaining live pups died within 6 hours after birth for 10 mg/kg PFOA group; 16% of neonates died in 5 mg/kg PFOA group.

Table S6: Characteristics of Hines et al. 2009 (study ID 260).

Study Element	Description
Methods	Mouse developmental toxicological study
Participants	Wild-type CD-1 mice<br>Timed-pregnant GD0 animals obtained from supplier<br>Total number of dams allocated: 85
Exposure	Dams treated with PFOA, ammonium salt (CAS# 3825-26-1) via daily gavage from GD1 to GD17.<br>Exposure groups:<br>• 5 dose groups = 0.01, 0.1, 0.3, 1, 5 mg PFOA/kg body weight/day; 15, 15, 15, 15, 10 dams for 0.01, 0.1, 0.3, 1, 5 mg PFOA/kg body weight/day groups, respectively.<br>• 1 control group = distilled water; 15 dams
Outcomes	1. Birth weight (g) – individual pups weighed at PND1 (day of birth or birth occurred previous night).<br>Number of dams analyzed:<br>• 10, 15, 15, 13, 14, 8 for control, 0.01, 0.1, 0.3, 1, 5 mg/kg PFOA groups, respectively<br>Not included in review: maternal weight; offspring weight gain; offspring glucose, leptin, and insulin levels; offspring late life organ and body weight; offspring fat to lean ratio; offspring feed consumption; effect of ovariectomy
Notes	Author responded to requests for additional information. Raw data provided by study author. Litter sizes were statistically equal across doses and control groups.

Table S7: Characteristics of Fenton et al. 2009 (study ID 264).

<table>
<tr><th>Study Element</th><th>Description</th></tr>
<tr><td>Methods</td><td>Mouse pharmacokinetic study</td></tr>
<tr><td>Participants</td><td>Wild-type CD-1 mice
Timed-pregnant GD14 animals obtained from supplier
Total number of dams allocated: 100</td></tr>
<tr><td>Exposure</td><td>Experimental groups:
Prenatal time point: dams treated with PFOA, ammonium salt (CAS# 3825-26-1), via single gavage on GD17.
Postnatal time point: dams treated with PFOA, ammonium salt (CAS# 3825-26-1), via single gavage on GD17.
Exposure groups:
3 dose groups = 0.1, 1, 5 mg PFOA/kg body weight
Prenatal time point: 5 dams/dose
Postnatal time point: 5 dams/dose
1 control group = deionized water
Prenatal time point: 5 dams
Postnatal time point: 5 dams</td></tr>
<tr><td>Outcomes</td><td>1. Fetal weight (g) – one individual fetus from each litter weighed at GD18.
Number of dams analyzed:
5, 5, 5, 4 for control, 0.1, 1, 5 mg/kg PFOA groups, respectively
2. Birth weight (g) – one individual pup from each litter weighed at PND1 (day of birth or birth occurred previous night).
Number of dams analyzed:
5, 4, 4, 5 for control, 0.1, 1, 5 mg/kg PFOA groups, respectively
Not included in review: PFOA serum concentration prior to birth; dam PFOA serum concentrations; pup PFOA serum concentrations</td></tr>
<tr><td>Notes</td><td>Author responded to requests for additional information. Raw data provided by study author. Number of live fetuses and litter sizes at birth were statistically equal across doses and control groups.</td></tr>
</table>

Table S8: Characteristics of White et al. 2009 (study ID 312).

Study Element	Description
Methods	Mouse developmental toxicological study
Participants	Wild-type CD-1 mice<br>Timed-pregnant GD0 animals obtained from supplier<br>Total number of dams allocated: 112
Exposure	Dams treated with PFOA, ammonium salt (CAS# 3825-26-1) via daily gavage from GD8 to GD17. At birth, pups were cross-fostered to obtain the following groups: 1) never exposed, 2) exposed in utero and via lactation, 3) exposed only in utero, 4) exposed only via lactation.<br>Exposure groups:<br>• 1 dose groups = 5 mg PFOA/kg body weight/day; 56 dams<br>• 1 control group = deionized water; 56 dams
Outcomes	1. Birth weight (g) – 3 individual female pups from each litter weighed at PND1 (at least 12 hours after birth and cross-foster); only 2 groups of pups relevant for review: 1) never exposed, 2) exposed in utero and via lactation.<br>Number of dams analyzed:<br>• 4 for each exposure group<br>Not included in review: 2 additional studies - late-life effects cross-foster study and restricted-exposure study; mammary gland development scores; circulating serum PFOA concentration; mammary gland differentiation in offspring; serum PFOA dosimetry
Notes	Author responded to requests for additional information. Raw data provided by study author.<br>Litter sizes were statistically equal across doses and control groups.

Table S9: Characteristics of Abbott et al. 2007 (study ID 528).

Study Element	Description
Methods	Mouse developmental toxicological study
Participants	Wild-type 129S1/SvlmJ mice<br>In-house breeding protocol<br>Total number of dams allocated: 157
Exposure	Dams treated with PFOA, ammonium salt (CAS# 3825-26-1), via daily gavage from GD1 to GD17.<br>Exposure groups:<br>• 7 dose groups = 0.1, 0.3, 0.6, 1, 5, 10, 20 mg PFOA/kg body weight/day; 15, 16, 26, 25, 12, 11, 16 dams for 0.1, 0.3, 0.6, 1, 5, 10, 20 mg PFOA/kg body weight/day groups, respectively<br>• 1 control group = deionized water; 36 dams
Outcomes	1. Birth weight (g) – pups grouped by sex and weighed at PND1 (day of birth or birth occurred previous night).<br>Number of dams analyzed:<br>• 19, 10, 7, 9, 13 for 0.1, 0.3, 0.6, 1 mg/kg PFOA groups, respectively<br>Not included in review: studies of PPARα knockout mouse model; maternal weight and reproductive outcomes; liver weight in dams and offspring; offspring survival, development, and growth; serum PFOA in dams and offspring
Notes	Author responded to requests for additional information. Raw data provided by study author. Study authors noted that the 129S1/SvlmJ strain is more sensitive to PFOA exposure than other strains, such as CD-1 strain mice. Litter sizes were statistically equal across dose groups up to 1 mg/kg PFOA and control groups; incidence of full litter resorption was statistically significantly higher in 5 mg/kg PFOA groups (83, 80, 100% of dams had full litter resorption for 5, 10, 20 mg/kg PFOA groups, respectively).

Table S10: Characteristics of White et al. 2007 (study ID 566).

**Study Element**	**Description**
Methods	Mouse developmental toxicological study
Participants	Wild-type CD-1 mice<br>Timed-pregnant GD0 animals obtained from supplier<br>Total number of dams allocated: 60
Exposure	Dams treated with PFOA, ammonium salt (CAS# 3825-26-1) via daily gavage for following time periods during pregnancy: GD1-17, GD8-17, GD12-17.<br>Exposure groups:<br>• 1 dose groups = 5 mg PFOA/kg body weight/day; 14, 16, 16 dams for GD1-17, GD8-17, GD12-17 time periods, respectively<br>• 1 control group = deionized water; 14 dams for GD1-17 time period
Outcomes	1. Birth weight (g) – individual pups weighed at PND1 (day of birth or birth occurred previous night).<br>Number of dams analyzed:<br>• 5 mg /kg PFOA: 6, 11, 10 for GD1-17, GD8-17, GD12-17 time periods, respectively<br>• Control: 10 for GD1-17 time periods<br>Not included in review: maternal weight gain; dam lactating mammary gland development; milk protein gene expression; blood PFOA concentrations; offspring mammary gland development.
Notes	Author responded to requests for additional information. Raw data provided by study author.<br>Litter sizes and number of uterine implantation sites were statistically equal across doses and control groups.

Table S11: Characteristics of Wolf et al. 2007 (study ID 571).

Study Element	Description
Methods	Mouse developmental toxicological study
Participants	Wild-type CD-1 mice<br>Timed-pregnant GD0 animals obtained from supplier<br>Total number of dams allocated: 182
Exposure	Experimental Groups<br>• Cross-foster: dams treated with PFOA, ammonium salt (CAS# 3825-26-1) via daily gavage from GD1-GD17.<br>• Windows of sensitivity: dams treated with PFOA, ammonium salt (CAS# 3825-26-1) via daily gavage for following time periods during pregnancy: GD7-17, GD10-17, GD13-17, GD15-17.<br>Exposure groups:<br>• 3 dose groups = 3, 5, 20 mg PFOA/kg body weight/day<br>Cross-foster: 28, 36 dams for 3, 5 mg PFOA/kg body weight/day groups, respectively<br>Windows of sensitivity:<br>5 mg PFOA/kg body weight/day: 14, 14, 12, 12 dams for GD7-17, GD10-17, GD13-17, GD15-17 time periods, respectively<br>20 mg PFOA/kg body weight/day: 6 dams for GD15-17 time period<br>• 1 control group = deionized water<br>Cross-foster: 48 dams<br>Windows of sensitivity: 12 dams for GD7-17 time period
Outcomes	1. Birth weight (g) – pups grouped by sex and weighed at birth (birth monitored at time intervals throughout night).<br>Number of dams analyzed:<br>• Control: 38, 7 for GD1-17, GD7-17 time points, respectively<br>• 3 mg/kg PFOA: 24 for GD1-17 time point<br>• 5 mg/kg PFOA: 25, 13, 13, 10, 10 for GD1-17, GD7-17, GD10-17, GD13-17, GD15-17 time points, respectively<br>• 20 mg/kg PFOA: 3 for GD15-17 time point<br>Not included in review: maternal weight; dam reproductive outcomes; dam liver weight; foster offspring survival, development, and growth; dam and offspring serum PFOA levels.
Notes	Author responded to requests for additional information. Raw data provided by study author. Litter sizes were statistically equal across doses and control groups. Incidence of whole litter loss was statistically significantly increased in the 5 mg/kg PFOA group as compared to other treatment groups (not caused by litter resorptions as statistically equal number of uterine implantation sites as compared to control).

Table S12: Characteristics of Lau et al. 2006 (study ID 635).

Study Element	Description
Methods	Mouse developmental toxicological study
Participants	Wild-type CD-1 mice<br>Timed-pregnant GD0 animals obtained from supplier<br>Total number of dams allocated: Unclear
Exposure	Dams treated with PFOA, ammonium salt (CAS# 3825-26-1) via daily gavage from GD1-GD17.<br>Exposure groups:<br>• 6 dose groups = 1, 3, 5, 10, 20, 40 mg PFOA/kg body weight/day<br>• 1 control group = water
Outcomes	1. Fetal weight (g) – fetuses weighed individually at GD18.<br>Number of dams analyzed:<br>• 45, 17, 17, 27, 26, 42, 9 dams for 0, 1, 3, 5, 10, 20, 40 mg/kg PFOA groups, respectively.<br>2. Birth weight (g) – pups weighed as litter at birth (birth monitored hourly).<br>Number of dams analyzed:<br>• 24, 8, 8, 30, 26, 7 for 0, 1, 3, 5, 10, 20 mg/kg PFOA groups, respectively.<br>Not included in review: serum PFOA compared for rats and mice; maternal weight gain; dam reproductive outcomes; fetal teratology; effect on time to parturition; offspring survival; offspring weight gain; offspring developmental landmarks; benchmark dose estimates.
Notes	Author responded to requests for additional information. Raw data provided by study author.<br>Full litter resorptions were statistically significantly increased in 5mg/kg PFOA and higher doses (100% litter resorption in 40 mg/kg PFOA group). Litter size at birth was statistically significantly decreased in 20 mg/kg PFOA group.

Table S13: Characteristics of Hinderliter et al. 2005 (study ID 711).

Study Element	Description
Methods	Rat pharmacokinetic study
Participants	Wild-type Sprague-Dawley rats<br>Timed-pregnant GD1 animals obtained from supplier<br>Total number of dams allocated: 20
Exposure	Dams treated with PFOA, ammonium salt (CAS# 3825-26-1), via daily gavage from GD4-GD21.<br>Exposure groups:<br>• 3 dose groups = 3, 10, 30 mg PFOA/kg body weight/day; 20 dams for each dose group<br>• 1 control group = deionized water; 20 dams
Outcomes	1. Birth weight (g) – pups grouped by sex and weighed at PND1 (day when birth complete).<br>Number of dams analyzed:<br>• 5 dams for each dose group<br>Not included in review: mortality; dam weight gain; offspring growth and survival; dam and offspring PFOA concentration
Notes	Author responded to requests for additional information. Published study does not present data on this outcome; authors provided full industry report performed according to good laboratory practices (GLP) that included data for birth weight. Litter sizes were statistically equal across doses and control groups.

Table S14: Characteristics of Staples et al. 1984 (study ID 1871).

<table>
<tr><th>Study Element</th><th>Description</th></tr>
<tr><td>Methods</td><td>Rat developmental toxicological study</td></tr>
<tr><td>Participants</td><td>Wild-type Sprague-Dawley rats
In-house breeding protocol
Total number of dams allocated: 224</td></tr>
<tr><td>Exposure</td><td>Experimental groups:
Inhalation: dams treated with PFOA, ammonium salt (CAS# 3825-26-1) via daily inhalation as a dust for 6 hour period from GD6-15; prenatal and postnatal time points.
Gavage: dams treated with PFOA, ammonium salt (CAS# 3825-26-1) via daily gavage from GD6-15; prenatal and postnatal time points.
Exposure groups:
4 inhalation dose groups = 0.1, 1, 10, 25 mg PFOA/m^3
Prenatal time point: 24, 24, 15, 12 dams for 0.1, 1, 10, 25 mg PFOA/m^3 groups, respectively
Postnatal time point: 12, 12, 6, 12 dams for 0.1, 1, 10, 25 mg PFOA/m^3 groups, respectively
3 inhalation control groups = in-house air only, in-house air pair-fed to 10 mg/m^3 dose group, in-house air pair-fed to 25 mg/m^3 dose group
Prenatal time point: 24, 6, 6 dams for in-house air only, pair-fed to 10 mg/m^3, and pair-fed to 25 mg/m3 groups, respectively
Postnatal time point: 18 dams for in-house air only
1 gavage dose group = 100 mg PFOA/kg body weight/day
Prenatal time point: 25 dams
Postnatal time point: 12 dams
1 gavage control group = stripped corn oil
Prenatal time point: 25 dams
Postnatal time point: 12 dams</td></tr>
<tr><td>Outcomes</td><td>1. Fetal weight (g) – individual fetuses weighed at GD21.
Number of dams analyzed:
Inhalation: 23, 24, 23, 15, 7, 6, 5 for 0, 0.1, 1, 10, 25 mg PFOA/m^3, pair-fed to 10 mg/m^3, pair-fed to 25 mg/m^3 groups, respectively.
Gavage: 24, 22 for 0, 100 mg/kg PFOA groups, respectively.
2. Birth weight (g) – individual pups weighed at PND1 (day of birth).
Number of dams analyzed:
Inhalation: 18, 10, 11, 6, 9 for 0, 0.1, 1, 10, 25 mg PFOA/m^3 groups, respectively.
Gavage: 12, 9 for 0, 100 mg/kg PFOA groups, respectively.
Not included in review: fetal teratology; dam body and liver weights; offspring survival; dam survival; offspring defects.</td></tr>
<tr><td>Notes</td><td>Study authors contacted to provide additional information, but information could not be obtained due to length of time since study conducted. Incidence of litter resorptions and litter size at birth were statistically equal across doses and control groups.</td></tr>
</table>

Table S15: Characteristics of Boberg et al. 2008 (study ID 3061).

Study Element	Description
Methods	Rat metabolic developmental toxicological study
Participants	Wild-type Wistar rats<br>Timed-pregnant GD3 animals obtained from supplier<br>Total number of dams allocated: 18
Exposure	Dams treated with PFOA (CAS# 333-67-1) via daily gavage from GD7-GD20/21.<br>Exposure groups:<br>• 1 dose groups = 20 mg PFOA/kg body weight/day; 8 dams<br>• 1 control group = corn oil; 10 dams
Outcomes	1. Fetal weight (g) – fetuses weighed individually at GD20/21.<br>Number of dams analyzed:<br>• 5, 6 for 0, 20 mg/kg PFOA groups, respectively.<br>Not included in review: outcomes for treatment with diisobutyl phthalate and butylparaben; steroid hormone measurement; plasma levels of metabolic chemicals; mRNA expression; P450c17 and PPARγ protein levels in testes.
Notes	Author responded to requests for additional information. Raw data provided by study author; published study does not present data on this outcome. Author noted that "some animals were sacrificed one day too early for their age. Therefore, in the GD21 group, about one fourth are GD20 and three fourths are GD21". Study did not discuss fetal mortality.

Table S16: Characteristics of Onishchenko et al. 2011 (study ID 3610).

Study Element	Description
Methods	Mouse neurobehavioral developmental toxicological study
Participants	Wild-type C57BL/6 mice<br>In-house breeding protocol<br>Total number of dams allocated: 16
Exposure	Dams treated with PFOA applied to food from GD1-GD20.<br>Exposure groups:<br>• 1 dose groups = 0.3 mg PFOA/kg body weight/day; 10 dams<br>• 1 control group = food applied with ethanol; 6 dams
Outcomes	1. Birth weight (g) – pups weighed at PND 1 (not clearly defined).<br>Number of dams analyzed:<br>• 6, 9 for 0, 0.3 mg/kg PFOA groups, respectively.<br>Not included in review: outcomes from treatment with PFOS; PFOA concentrations in tissues; locomotor and exploratory activity; circadian activity; anxiety-related behavior; depression-like behavior; muscle strength; motor coordination
Notes	Author responded to requests for additional information. Raw data provided by study author; published study does not present data on this outcome. The author reported results for a larger number of PFOA-treated dams than originally allocated in the paper. Results from the study author do not clearly explain if the body weights are litter averages. Failure to report pertinent study details resulted in exclusion from quantitative analysis for birth weight outcome, despite meeting inclusion criteria otherwise. Authors did not specify chemical form of PFOA (assume CAS# 335-67-1). Litter sizes were statistically equal across doses and control groups.

Table S17: Characteristics of White et al. 2011 (study ID 3862).

Study Element	Description
Methods	Mouse multigenerational developmental toxicological study
Participants	Wild-type CD-1 mice<br>Timed-pregnant GD0 animals obtained from supplier<br>Total number of dams allocated: 33
Exposure	Dams treated with PFOA, ammonium salt (CAS# 3825-26-1) via daily gavage from GD1 to GD17.<br>Exposure groups:<br>• 2 dose groups = 1, 5 mg PFOA/kg body weight/day; 12, 11 dams for 1, 5 mg PFOA/kg body weight/day groups, respectively.<br>• 1 control group = deionized water; 10 dams
Outcomes	1. Birth weight (g) – F1 pups grouped by sex and weighed at PND1 (day of birth or birth occurred previous night).<br>Number of dams analyzed:<br>• 18, 23, 19 dams for 0, 1, 5 mg/kg PFOA groups, respectively.<br>Not included in review: results for F2 generation; dam weight gain; offspring growth and survival; mammary development; lactation success; water consumption; serum PFOA
Notes	Author responded to requests for additional information. Raw data provided by study author; published study does not present data on this outcome. Published study included groups treated with drinking water containing PFOA, but study authors did not provide data for these studies and stated that only the groups treated via gavage could be compared. The number of uterine implantation sites was statistically equal across doses and control groups. Litter size at birth and prenatal survival were statistically significantly decreased in 5 mg/kg PFOA groups.

Table S18: Characteristics of York 2002 (study ID 5122).

Study Element	Description
Methods	Rat multigenerational developmental toxicological study
Participants	Wild-type Sprague-Dawley rats<br>Virgin animals obtained from supplier<br>Total number of dams allocated: 150
Exposure	Dams treated with PFOA, ammonium salt (CAS# 3825-26-1) via daily gavage from 70 days prior to breeding through lactation.<br>Exposure groups:<br>• 4 dose groups = 1, 3, 10, 30 mg PFOA/kg body weight/day; 30 dams for each dose group<br>• 1 control group = deionized water; 30 dams
Outcomes	1. Birth weight (g) – F1 pups weighed individually at PND1 (day of birth).<br>Number of dams analyzed:<br>• 28, 27, 29, 29, 28 for 0, 1, 3, 10, 30 mg/kg PFOA groups, respectively<br>Not included in review: duration of gestation; fertility index; number/sex offspring per litter; number of implantation sites; maternal behavior; necropsy for gross lesions; organ weight and evaluation
Notes	Author responded to requests for additional information. This study was an industry report performed according to good laboratory practices (GLP). The report supports papers in peer-reviewed literature. Litter sizes at birth were statistically equal across doses and control groups.

Table S19: Characteristics of Hagenaars et al. 2011 (study ID 59).

Study Element	Description
Methods	Zebrafish (*Danio rerio*) developmental toxicological study
Participants	Wild-type zebrafish eggs<br>In-house breeding protocol<br>Eggs collected 30 min after spawning<br>Total number of eggs allocated: 368
Exposure	Eggs immersed in PFOA (CAS# 335-67-1) test solutions within 60 min after spawning and continuously treated until 120 hours post fertilization (hpf). Hatching typically occurs at 72 hpf.<br>Exposure Groups:<br>• 8 dose groups = 15, 20, 30, 40, 50, 75, 100, 250 mg PFOA/L freshwater; 40 eggs each<br>• 1 control group = freshwater; 48 eggs
Outcomes	1. Length (mm) - individual zebrafish embryos measured at 120 hpf. Number of zebrafish embryos analyzed:<br>• 34, 36, 32, 33, 30, 33, 33, 31, 30 for control, 15, 20, 30, 40, 50, 75, 100, 250 mg PFOA/L freshwater groups, respectively<br>Not included in review: outcomes for treatment with PFOS, PFBS, and PFBA; concentration-response relationship; malformations; hatching rate; heart rate; correlation heart rate and length.
Notes	Author responded to requests for additional information. Raw data provided by study author (including 15, 20 mg PFOA/L doses not presented in paper). PFOA induced mortality at highest doses in dose-dependent manner (calculated EC_{50} 205.72 mg PFOA/L). Embryo hatching statistically significantly delayed in 50 mg PFOA/L and higher dose groups.

Table S20: Characteristics of Wang et al. 2010 (study ID 86).

**Study Element**	**Description**
Methods	Fruit fly (*Drosophila melanogaster)* developmental toxicological study
Participants	Wild-type flies (W118 stock)<br>In-house breeding protocol<br>Total number of female flies allocated (larvae stage): 300<br>Total number of female flies allocated (pupae stage): Unclear
Exposure	Experimental groups:<br>• Larvae: one-day-old female flies allowed to lay eggs for 2 hours in vials containing food with PFOA, ammonium salt (CAS# 3825-26-1). Eggs hatched and developed on media and larvae were collected at 30, 48, 72, 96, and 110 hours after egg laying (AEL).<br>• Pupae: same regimen as above. Larvae allowed to develop to white pupae stage.<br>Exposure groups:<br>• 2 dose groups = 100, 500 µM PFOA in food<br>Larvae group: 20 female flies/dose, for each time point<br>Pupae group: Unclear how many female flies originally allocated<br>• 1 control group = untreated food<br>Larvae group: 20 female flies for each time point<br>Pupae group: Unclear how many female flies originally allocated
Outcomes	1. Larval volume (arbitrary units) – calculated based on individual measurements of length and diameter.<br>Number of larvae analyzed:<br>• Control: 10, 23, 30, 31, 28 for 30, 48, 72, 96, 110 AEL groups, respectively<br>• 100 µM PFOA in food: 15, 20, 26, 33, 33 for 30, 48, 72, 96, 110 AEL groups, respectively<br>• 500 µM PFOA in food: 14, 15, 46, 25, 29 for 30, 48, 72, 96, 110 AEL groups , respectively<br>2. Pupae weight (g) – individual pupae weights.<br>Number of pupae analyzed:<br>• 38, 35, 25 for control, 100, 500 µM PFOA in food groups, respectively<br>Not included in review: lifespan, behavior, numbers of emerging progeny, lethality, developmental progress, effects of nutrient supplementation
Notes	Author responded to requests for additional information. Raw data provided by study author (including length measurements plotted for analysis). Emergence of progeny was statistically significantly reduced in both PFOA dose groups (24% and 73% decrease compared to control group for 100, 500 µM PFOA in food groups, respectively).

Table S21: Characteristics of Pinkas et al. 2010 (study ID 187).

Study Element	Description
Methods	Chicken neurodevelopmental toxicological study
Participants	Cobb I chicken broiler strain (*Gallus gallus domesticus*)<br>Fertile heterogeneous stock eggs obtained from supplier<br>Total number of eggs allocated: Unclear
Exposure	Eggs injected with PFOA (CAS# 335-67-1) at incubation day 0.<br>Exposure groups:<br>• 2 dose groups = 5, 10 mg PFOA/kg egg<br>• 1 control group = saline
Outcomes	1. Hatchling weight (g) – individual hatchlings weighed 24 hours after hatching.<br>Number of hatchlings analyzed:<br>• 30, 12, 10 for control, 5, 10 mg PFOA/kg egg groups, respectively<br>Not included in review: outcomes from treatment with PFOS; hatching and survival; morphological and functional scores; imprinting scores; protein kinase C concentrations in the intermedial part of the hyperstriatum ventrale
Notes	Author responded to requests for additional information. Study author provided data estimates used to create figure in paper. On incubation day 19, survival of embryos was statistically significantly reduced (approx 45-55%) in the PFOA treated groups as compared to controls; hatching was statistically significantly reduced (70-80%) compared to controls.

Table S22: Characteristics of O'Brien et al. 2009 (study ID 236).

Study Element	Description
Methods	Chicken developmental toxicological study
Participants	White leghorn chicken (*Gallus gallus domesticus*)<br>Eggs obtained from supplier<br>Total number of eggs allocated: 120
Exposure	Eggs injected with PFOA at incubation day 0.<br>Exposure groups:<br>• 4 dose groups = 0.01, 0.1, 1, 10 mg PFOA/kg egg; 20 eggs for each dose group<br>• 2 control groups = uninjected, DMSO; 20 eggs for each control group
Outcomes	1. Embryo weight (g) – individual embryos weighed at pipping star or day 22, whichever came first.<br>Number of embryos analyzed:<br>• 12, 18, 17, 15, 16, 15 for uninjected, DMSO only, 0.01, 0.1, 1, 10 mg PFOA/kg egg egg groups, respectively<br>Not included in review: outcomes from treatment with PFUdA and PFDS; pipping success; hepatic PFC concentrations; mRNA expression
Notes	Author responded to requests for additional information. Raw data provided by study author; published study does not present data on this outcome. Authors did not specify chemical form of PFOA (assume CAS# 335-67-1). Pipping success and developmental stage at embryo death were statistically equal across doses and control groups.

Table S23: Characteristics of Jiang et al. 2012 (study ID 3926).

Study Element	Description
Methods	Chicken developmental toxicological study
Participants	Chicken (*Gallus gallus*)<br>Fertile stock eggs obtained from supplier<br>Total number of eggs allocated: 176
Exposure	Experimental groups:<br>• Embryonic time point: eggs injected with PFOA at incubation day 0.<br>• Hatchling time point: eggs injected with PFOA at incubation day 0.<br>Exposure groups:<br>• 3 dose groups = 0.5, 1, 2 mg PFOA/kg egg<br>Embryonic time point: 10, 12, 9 eggs for 0.5, 1, 2 mg PFOA/kg egg groups, respectively.<br>Hatchling time point: 22, 23, 24 eggs for 0.5, 1, 2 mg PFOA/kg egg groups, respectively.<br>• 2 control groups = uninjected, sunflower oil<br>Embryonic time point: 10, 9 eggs for uninjected, sunflower oil groups, respectively.<br>Hatchling time point: 36, 21 eggs for uninjected, sunflower oil groups, respectively.
Outcomes	1. Yolk free body weight (g)<br>Embryonic time point: individual embryos removed from eggs and weighed at embryonic day 19.<br>Number of embryos analyzed:<br>• 9, 8, 7, 9, 7 for uninjected, sunflower oil only, 0.5, 1, 2 mg PFOA/kg egg groups, respectively<br>Hatchling time point: individual hatchlings weighed 16-24 hours after hatching.<br>Number of hatchlings analyzed (same hatchlings used to examine crown to rump length outcome described below):<br>• 26, 10, 11, 12, 9 for uninjected, sunflower oil only, 0.5, 1, 2 mg PFOA/kg egg groups, respectively<br>2. Crown to rump length (mm)<br>Hatchling time point: individual hatchlings measured 16-24 hours after hatching - see above for number of hatchlings analyzed.<br>Not included in review: embryo and hatchling heart weight; embryo and hatchling liver weight; embryo and hatchling mortality; hatchability; embryo cardiac morphology; hatchling cardiac ultrasound; hatchling cardiac myofibril ATPase; hatchling serum PFOA concentration
Notes	Author responded to requests for additional information. Raw data provided by study author. Authors did not specify chemical form of PFOA (assume CAS# 335-67-1). In embryos, mortality was statistically significantly increased in 2 mg PFOA/kg egg group (76% increase compared to control). In hatchlings, mortality and hatching were statistically equal across doses and control groups.

Table S24: Characteristics of Spachmo and Arukwe 2012 (study ID 3932).

Study Element	Description
Methods	Salmon endocrine and developmental toxicological study
Participants	Atlantic salmon (*Salmo salar*)<br>Eggs obtained from supplier<br>Total number of eggs allocated: Unclear
Exposure	Eggs exposed to PFOA in water through day 48. Hatching occurred at day 20. Larvae were collected at days 21, 35, 49, and 56.<br>Exposure groups:<br>• 1 dose groups = 100 μg PFOA/L water<br>• 1 control group = water with carrier solvent (methanol)
Outcomes	1. Length (cm) – larvae measured using microscope with ruler and digital camera.<br>Number of larvae analyzed (same larvae used to examine dry weight outcome described below):<br>• 10 randomly selected larvae for each time point and dose group<br>2. Dry weight (g) – larvae dried in heat cabinet and weighed using a micro-weight scale - see above for number of larvae analyzed.<br>Not included in review: outcomes from treatment with PFOS; bone development; effects on HTP-axis; effects on ER expression; effects on GH-IGF axis; effects on chondrogenic and osteogenic pathways
Notes	Authors did not respond to requests for additional information. Authors did not specify chemical form of PFOA (assume CAS# 335-67-1). Data estimates for figures presented in the published paper were obtained using an online digital ruler. Study did not discuss survival.

Table S25: Risk of bias summary of Hu et al. 2010 (study ID 68).

Bias domain	Authors' judgment	Support for judgment
Sequence generation	Probably high risk	Randomization scheme not discussed; adequate randomization is not standard protocol for these types of experiments.
Allocation concealment	Probably high risk	Allocation concealment not discussed; adequate concealment is not standard protocol for these types of experiments.
Blinding	Probably high risk	Blinding not discussed; adequate blinding is not standard protocol for these types of experiments.
Incomplete outcome data	Low risk	Based on author response, dams and offspring were adequately followed; author reported numbers allocated.
Selective reporting	Low risk	Outcomes outlined in abstract/methods section of paper were reported in results section; "n" provided by author; raw data provided by author.
Conflict of interest	Probably low risk	Associated funds and persons appear to be from government and/or academia only and free of financial interests in study results. However, no claim denying conflicts of interest was made.
Other bias	Low risk	No other potential biases are suspected.

Table S26: Risk of bias summary of Yahia et al. 2010 (study ID 103).

**Bias domain**	**Authors' judgment**	**Support for judgment**
Sequence generation	Probably high risk	Randomization scheme not discussed; adequate randomization is not standard protocol for these types of experiments.
Allocation concealment	Probably high risk	Allocation concealment not discussed; adequate concealment is not standard protocol for these types of experiments.
Blinding	Probably high risk	Blinding not discussed; adequate blinding is not standard protocol for these types of experiments.
Incomplete outcome data	Probably high risk	Dams were not adequately followed; offspring mortality reported; numbers allocated not reported.
Selective reporting	Probably high risk	Neonatal weight outcome reported in results section of paper, but not pre-specified in methods section; time point of weight measurement not reported; adequate "n" reported for neonate outcomes; average values reported for fetal outcome so can calculate "n" analyzed.
Conflict of interest	Probably low risk	Funding source not stated, but associated persons appear to be from government and/or academia only and free of financial interests in study results. However, no claim denying conflicts of interest was made.
Other bias	Low risk	No other potential biases are suspected.

Table S27: Risk of bias summary of Hines et al. 2009 (study ID 260).

**Bias domain**	**Authors' judgment**	**Support for judgment**
Sequence generation	Probably low risk	Mice were "randomly distributed among treatment groups", although details of random sequence generation were not discussed.
Allocation concealment	Probably high risk	Allocation concealment not discussed; adequate concealment is not standard protocol for these types of experiments.
Blinding	Probably high risk	Blinding not discussed; adequate blinding is not standard protocol for these types of experiments.
Incomplete outcome data	Probably low risk	Based on author response, dams were adequately followed; offspring mortality not reported, but noted that losses equivalent between groups; author reported numbers allocated.
Selective reporting	Probably low risk	Author reported different numbers allocated than reported in paper and noted that a figure in the paper displayed male and female weights even though the legend specifies female weights only; "n" provided by author; raw data provided by author.
Conflict of interest	Probably high risk	Associated funds and persons appear to be from government and/or academia only and free of financial interests in study results. However, acknowledgement was made to employee from company financially invested in PFOA (Dow) for "constructive input on this manuscript" and no claim denying conflicts of interest was made.
Other bias	Low risk	No other potential biases are suspected.

Table S28: Risk of bias summary of Fenton et al. 2009 (study ID 264).

Bias domain	Authors' judgment	Support for judgment
Sequence generation	Probably low risk	Mice were "were weighed and randomly distributed among PFOA treatment groups", although details of random sequence generation were not discussed.
Allocation concealment	Probably high risk	Allocation concealment not discussed; adequate concealment is not standard protocol for these types of experiments.
Blinding	Probably high risk	Blinding not discussed; adequate blinding is not standard protocol for these types of experiments.
Incomplete outcome data	Probably high risk	Based on author response, offspring mortality not assessed; dams were adequately followed; weighed one pup per litter; numbers allocated reported.
Selective reporting	Probably low risk	Author reported that one pup per litter included in weight outcome, but this was not clearly stated in paper; "n" provided by author; raw data provided by author.
Conflict of interest	Low risk	Funding source not stated, but associated persons appear to be from government and/or academia only and free of financial interests in study results. "The author declares that there are no conflicts of interest."
Other bias	Low risk	No other potential biases are suspected.

Table S29: Risk of bias summary of White et al. 2009 (study ID 312).

Bias domain	Authors' judgment	Support for judgment
Sequence generation	Probably low risk	"Upon arrival at the animal facility on GD 0, mice were weighed and randomly assigned to one of two treatment groups", although details of random sequence generation were not discussed.
Allocation concealment	Probably high risk	Allocation concealment not discussed; adequate concealment is not standard protocol for these types of experiments.
Blinding	Probably high risk	Blinding not discussed; adequate blinding is not standard protocol for these types of experiments.
Incomplete outcome data	Probably high risk	Based on author response, offspring were adequately followed; only reported on results from 3 pups weighed from each of 4 dams (56 allocated/group); numbers allocated reported.
Selective reporting	Low risk	Outcomes outlined in abstract/methods section of paper were reported in results section; adequate "n" reported; raw data provided by author.
Conflict of interest	Probably high risk	Associated funds and persons appear to be from government and/or academia only and free of financial interests in study results. "The authors declare that there are no conflicts of interest." However, company financially invested in PFOA (3M) provided analysis of PFOA chemical used in study.
Other bias	Low risk	No other potential biases are suspected.

Table S30: Risk of bias summary of Abbott et al. 2007 (study ID 528).

**Bias domain**	**Authors' judgment**	**Support for judgment**
Sequence generation	Probably low risk	"Plug positive female mice were weighed, randomly assigned to treatment groups", although details of random sequence generation were not discussed.
Allocation concealment	Probably high risk	Allocation concealment not discussed; adequate concealment is not standard protocol for these types of experiments.
Blinding	Probably high risk	Blinding not discussed; adequate blinding is not standard protocol for these types of experiments.
Incomplete outcome data	Low risk	Based on author response, dams and offspring were adequately followed; author reported numbers allocated.
Selective reporting	Low risk	Outcomes outlined in abstract/methods section of paper were reported in results section; "n" provided by author; raw data provided by author.
Conflict of interest	Probably low risk	Funding source not stated, but associated persons appear to be from government and/or academia only and free of financial interests in study results. However, no claim denying conflicts of interest was made.
Other bias	Low risk	No other potential biases are suspected.

Table S31: Risk of bias summary of White et al. 2007 (study ID 566).

Bias domain	Authors' judgment	Support for judgment
Sequence generation	Probably low risk	"Animals were weighed upon arrival and randomly distributed among four treatment groups", although details of random sequence generation were not discussed.
Allocation concealment	Probably high risk	Allocation concealment not discussed; adequate concealment is not standard protocol for these types of experiments.
Blinding	Probably high risk	Blinding not discussed; adequate blinding is not standard protocol for these types of experiments.
Incomplete outcome data	Low risk	Based on author response, dams and offspring were adequately followed; numbers allocated reported.
Selective reporting	Low risk	Outcomes outlined in abstract/methods section of paper were reported in results section; "n" provided by author; raw data provided by author.
Conflict of interest	Probably low risk	Associated funds and persons appear to be from government and/or academia only and free of financial interests in study results. However, no claim denying conflicts of interest was made.
Other bias	Low risk	No other potential biases are suspected.

Table S32: Risk of bias summary of Wolf et al. 2007 (study ID 571).

**Bias domain**	**Authors' judgment**	**Support for judgment**
Sequence generation	Probably low risk	"Upon arrival at the animal facility, mice were weighed and randomly assigned", although details of random sequence generation were not discussed.
Allocation concealment	Probably high risk	Allocation concealment not discussed; adequate concealment is not standard protocol for these types of experiments.
Blinding	Probably high risk	Blinding not discussed; adequate blinding is not standard protocol for these types of experiments.
Incomplete outcome data	Low risk	Dams and offspring were adequately followed; numbers allocated reported.
Selective reporting	Low risk	Outcomes outlined in abstract/methods section of paper were reported in results section; "n" provided by author; raw data provided by author.
Conflict of interest	Probably high risk	Funding source not stated, but associated persons appear to be from government and/or academia only and free of financial interests in study results. However, company financially invested in PFOA (3M) provided analysis of PFOA chemical used in study and no claim denying conflicts of interest was made.
Other bias	Low risk	No other potential biases are suspected.

Table S33: Risk of bias summary of Lau et al. 2006 (study ID 635).

Bias domain	Authors' judgment	Support for judgment
Sequence generation	High risk	"Animals were randomly assigned to treatment groups". Author noted that mice were ranked according to weight at arrival then assigned evenly to each group at "random" but that a component such as a random number generator was not used in the sequence generation process.
Allocation concealment	Probably high risk	Allocation concealment not discussed; adequate concealment is not standard protocol for these types of experiments.
Blinding	Probably high risk	Blinding not discussed; adequate blinding is not standard protocol for these types of experiments.
Incomplete outcome data	Probably low risk	Dams and offspring were adequately followed; numbers allocated not reported.
Selective reporting	Probably low risk	Outcomes outlined in abstract/methods section of paper were reported in results section; adequate "n" provided by author for fetal and birth outcomes (pups per litter provided as range).
Conflict of interest	Probably high risk	Associated funds and persons appear to be from government and/or academia only and free of financial interests in study results. However, company financially invested in PFOA (3M) provided analysis of PFOA chemical used in study and no claim denying conflicts of interest was made.
Other bias	Low risk	No other potential biases are suspected.

Table S34: Risk of bias summary of Hinderliter et al. 2005 (study ID 711).

Bias domain	Authors' judgment	Support for judgment
Sequence generation	Probably low risk	"Dams were ranked on…body weights and assigned to control and experimental groups by random sampling from the ranked list...Rats in each group were then randomly assigned to each subset", although details of random sequence generation were not discussed.
Allocation concealment	Probably high risk	Allocation concealment not discussed; adequate concealment is not standard protocol for these types of experiments.
Blinding	Probably high risk	Blinding not discussed; adequate blinding is not standard protocol for these types of experiments.
Incomplete outcome data	Low risk	Based on author response, dams and pups were adequately followed; numbers allocated reported.
Selective reporting	Low risk	Weight outcome outlined in methods section of paper, but not reported in results section; however, detailed outcome data presented in report provided by author; "n" provided by author; raw data provided by author.
Conflict of interest	High risk	Du Pont and 3M sponsored study and authors were employed by companies.
Other bias	Low risk	No other potential biases are suspected.

Table S35: Risk of bias summary of Staples et al. 1984 (study ID 1871).

**Bias domain**	**Authors' judgment**	**Support for judgment**
Sequence generation	High risk	Females were ranked within breeding days by body weight and assigned to groups by rotating in order of rank.
Allocation concealment	High risk	Females were allocated by rotation.
Blinding	Probably low risk	"To limit possible bias in the examination of maternal and fetal specimens, the dams were coded (group designation unknown to examiner) from just before sacrifice until all maternal and fetal data were collected"; unclear if applies to birth outcomes.
Incomplete outcome data	Low risk	Dams and offspring were adequately followed; numbers allocated reported.
Selective reporting	Probably low risk	Outcomes outlined in abstract/methods section of paper were reported in results section; average values reported so can calculate "n" analyzed.
Conflict of interest	High risk	Du Pont sponsored study and authors were employed by the company.
Other bias	Low risk	No other potential biases are suspected.

Table S36: Risk of bias summary of Boberg et al. 2008 (study ID 3061).

Bias domain	Authors' judgment	Support for judgment
Sequence generation	Probably low risk	"The dams were randomized into seven groups of eight with similar body weight distributions", although details of random sequence generation were not discussed.
Allocation concealment	Probably high risk	Allocation concealment not discussed; adequate concealment is not standard protocol for these types of experiments.
Blinding	Probably high risk	Blinding not discussed; adequate blinding is not standard protocol for these types of experiments.
Incomplete outcome data	Low risk	Based on author response, dams and pups were adequately followed; numbers allocated reported.
Selective reporting	Probably low risk	Author reported different numbers allocated than reported in paper; weight outcome pre-specified in methods section of paper and reported as "data not shown" in results section; however, detailed outcome data provided by author; "n" provided by author; raw data provided by author.
Conflict of interest	Low risk	Associated funds and persons appear to be from government and/or academia only and free of financial interests in study results. Authors claim "none" for conflicts of interest.
Other bias	Low risk	No other potential biases are suspected.

Table S37: Risk of bias summary of Onishchenko et al. 2011 (study ID 3610).

Bias domain	Authors' judgment	Support for judgment
Sequence generation	Probably high risk	Randomization scheme not discussed; adequate randomization is not standard protocol for these types of experiments.
Allocation concealment	Probably high risk	Allocation concealment not discussed; adequate concealment is not standard protocol for these types of experiments.
Blinding	Probably high risk	Blinding not discussed; adequate blinding is not standard protocol for these types of experiments.
Incomplete outcome data	Probably high risk	Dams and offspring were not adequately followed, but noted "litter size and sex ration were similar in control and exposed groups"; number allocated reported, but author provided data for more dams than allocated.
Selective reporting	Probably high risk	Weight outcome reported in results section of paper, but not pre-specified in methods section; "n" provided by author, but unclear if pup number or dam number and greater than numbers allocated in paper; author provided raw data.
Conflict of interest	Probably low risk	Associated funds and persons appear to be from government and/or academia only and free of financial interests in study results. However, no claim denying conflicts of interest was made.
Other bias	Low risk	No other potential biases are suspected.

Table S38: Risk of bias summary of White et al. 2011 (study ID 3862).

**Bias domain**	**Authors' judgment**	**Support for judgment**
Sequence generation	Probably low risk	"Timed pregnant dams were randomly distributed among five treatment groups", although details of random sequence generation were not discussed.
Allocation concealment	Probably high risk	Allocation concealment not discussed; adequate concealment is not standard protocol for these types of experiments.
Blinding	Probably high risk	Blinding not discussed; adequate blinding is not standard protocol for these types of experiments.
Incomplete outcome data	Low risk	Dams and offspring were adequately followed; numbers allocated reported, but author provided different numbers allocated.
Selective reporting	Probably low risk	Author reported different numbers allocated than reported in paper; weight outcome outlined in methods section paper, but not reported in results section; however, detailed outcome data provided by author; "n" provided by author; raw data provided by author.
Conflict of interest	Low risk	Associated funds and persons appear to be from government and/or academia only and free of financial interests in study results. "The authors declare they have no actual or potential competing financial interests."
Other bias	Low risk	No other potential biases are suspected.

Table S39: Risk of bias summary of York 2002 (study ID 5122).

Bias domain	Authors' judgment	Support for judgment
Sequence generation	Low risk	"Upon arrival parental generation rats will be assigned to individual housing on the basis on computer-generated random units…The rats will be assigned to dosage groups based on computer-generated (weight-ordered) randomization procedures."
Allocation concealment	Probably high risk	Allocation concealment not discussed; adequate concealment is not standard protocol for these types of experiments.
Blinding	Probably high risk	Blinding not discussed; adequate blinding is not standard protocol for these types of experiments.
Incomplete outcome data	Low risk	Dams and offspring were adequately followed; numbers allocated reported.
Selective reporting	Low risk	Outcomes outlined in abstract/methods section of paper were reported in results section; adequate "n" reported.
Conflict of interest	High risk	3M sponsored study and authors were employed by company.
Other bias	Low risk	No other potential biases are suspected.

Table S40: Risk of bias summary of Hagenaars et al. 2011 (study ID 59).

Bias domain	Authors' judgment	Support for judgment
Sequence generation	Probably high risk	Randomization scheme not discussed; adequate randomization is not standard protocol for these types of experiments.
Allocation concealment	Probably high risk	Allocation concealment not discussed; adequate concealment is not standard protocol for these types of experiments.
Blinding	Probably high risk	Blinding not discussed; adequate blinding is not standard protocol for these types of experiments.
Incomplete outcome data	Low risk	Hatchability and mortality reported; numbers allocated reported.
Selective reporting	Probably low risk	Author reported different numbers allocated than reported in paper; "n" provided by author; raw data provided by author.
Conflict of interest	Probably low risk	Associated funds and persons appear to be from government and/or academia only and free of financial interests in study results. However, no claim denying conflicts of interest was made.
Other bias	Low risk	No other potential biases are suspected.

Table S41: Risk of bias summary of Wang et al. 2010 (study ID 86).

Bias domain	Authors' judgment	Support for judgment
Sequence generation	Probably low risk	"Cohorts of…flies were assigned randomly into vials"; unclear if applies to growth outcomes; details of random sequence generation were not discussed.
Allocation concealment	Probably high risk	Allocation concealment not discussed; adequate concealment is not standard protocol for these types of experiments.
Blinding	Probably high risk	Blinding not discussed; adequate blinding is not standard protocol for these types of experiments.
Incomplete outcome data	Probably high risk	Hatchability and mortality not reported; only subset of treatment group analyzed for outcome; numbers allocated reported.
Selective reporting	Probably low risk	Pupae weight outcome reported in results section of paper, but not pre-specified in methods section; "n" provided by author; raw data provided by author.
Conflict of interest	Probably low risk	Associated funds and persons appear to be from government and/or academia only and free of financial interests in study results. However, no claim denying conflicts of interest was made.
Other bias	Low risk	No other potential biases are suspected.

Table S42: Risk of bias summary of Pinkas et al. 2010 (study ID 187).

**Bias domain**	**Authors' judgment**	**Support for judgment**
Sequence generation	Probably high risk	Randomization scheme not discussed; adequate randomization is not standard protocol for these types of experiments.
Allocation concealment	Probably high risk	Allocation concealment not discussed; adequate concealment is not standard protocol for these types of experiments.
Blinding	Probably high risk	Blinding not discussed; adequate blinding is not standard protocol for these types of experiments.
Incomplete outcome data	Probably low risk	Hatchlings were adequately followed; numbers allocated not reported.
Selective reporting	Probably high risk	Weight outcome reported in results section paper, but not pre-specified in methods section; adequate "n" reported.
Conflict of interest	Low risk	Associated funds and persons appear to be from government and/or academia only and free of financial interests in study results. "No author has any conflict of interest to disclose."
Other bias	Low risk	No other potential biases are suspected.

Table S43: Risk of bias summary of O'Brien et al. 2009 (study ID 236).

Bias domain	Authors' judgment	Support for judgment
Sequence generation	Probably low risk	"Eggs were randomly distributed among 4 dose groups", although details of random sequence generation were not discussed.
Allocation concealment	Probably high risk	Allocation concealment not discussed; adequate concealment is not standard protocol for these types of experiments.
Blinding	Probably high risk	Blinding not discussed; adequate blinding is not standard protocol for these types of experiments.
Incomplete outcome data	Low risk	Based on author response, embryos were adequately followed; numbers allocated reported.
Selective reporting	Low risk	Weight outcome outlined in methods section paper, but not reported in results section; however, detailed outcome data provided by author; "n" provided by author; raw data provided by author.
Conflict of interest	Low risk	Associated funds and persons appear to be from government and/or academia only and free of financial interests in study results. "The authors declare that there are no conflicts of interest."
Other bias	Low risk	No other potential biases are suspected.

Table S44: Risk of bias summary of Jiang et al. 2012 (study ID 3926).

**Bias domain**	**Authors' judgment**	**Support for judgment**
Sequence generation	Probably high risk	"Eggs were...given ID numbers, and evenly distributed by weight among doses"; adequate randomization is not standard protocol for these types of experiments.
Allocation concealment	Probably low risk	Eggs "given ID numbers" before allocation so there may have been allocation concealment, but not explicitly stated.
Blinding	Probably low risk	Eggs were "given ID numbers" so blinding may have been applied, but not explicitly stated.
Incomplete outcome data	Probably high risk	Hatching and mortality reported, but authors noted mortality not carefully tracked for all eggs included in outcome measurement; author reported numbers allocated.
Selective reporting	Probably low risk	Weight and length outcomes reported in results section of paper, but not pre-specified in methods section; "n" provided by author; raw data provided by author.
Selective reporting	Probably low risk	Weight and length outcomes reported in results section of paper, but not pre-specified in methods section; "n" provided by author; raw data provided by author.
Conflict of interest	Low risk	Funding source not stated, but associated persons appear to be from government and/or academia only and free of financial interests in study results. "No conflicts of interest."
Other bias	Low risk	No other potential biases are suspected.

Table S45: Risk of bias summary of Spachmo and Arukwe 2012 (study ID 3932).

Bias domain	Authors' judgment	Support for judgment
Sequence generation	Probably high risk	Randomization scheme not discussed; adequate randomization is not standard protocol for these types of experiments.
Allocation concealment	Probably high risk	Allocation concealment not discussed; adequate concealment is not standard protocol for these types of experiments.
Blinding	Probably low risk	"All cardiac and bone measurements were performed blind with respect to treatment and sampling." Unclear if applies to weight outcome.
Incomplete outcome data	High risk	Mortality not reported; only subset of treatment group analyzed for outcome; numbers allocated not reported.
Selective reporting	Low risk	Outcomes outlined in abstract/methods section of paper were reported in results section; adequate "n" reported.
Conflict of interest	Probably low risk	Associated funds and persons appear to be from government and/or academia only and free of financial interests in study results. However, no claim denying conflicts of interest was made.
Other bias	Low risk	No other potential biases are suspected.

Table S46: 95% Confidence interval estimates for mammalian fetal weight measurements, for each tested dose of PFOA (see Figure 5A).

Source [source ID]	Species	Route of Exposure	PFOA dose[a]	Lower bound	Upper bound
Yahia et al. 2010 [103]	Mouse	Gavage	1*	0.03	0.09
			5*	-0.17	-0.11
			10*	-0.44	-0.38
Fenton et al. 2009 [264]	Mouse	Gavage	0.1	-0.08	0.44
			1	0.04	0.64
			5	-0.22	0.36
Lau et al. 2006 [635][b]	Mouse	Gavage	1	-0.14	0.00
			3	-0.11	0.07
			5	-0.11	0.07
			10*	-0.18	0.04
			20*	-0.41	0.03
Staples et al. 1984 [1871]	Rat	Gavage	100	0.02	0.38
Boberg et al. 2008 [3061]	Rat	Gavage	20	-0.85	1.35
Staples et al. 1984 [1871]	Rat	Inhalation	0.1 mg/m^3	-0.23	0.03
			1 mg/m^3	-0.21	0.01
			10 mg/m^3	-0.26	0.06
			25 mg/m^3*	-0.65	-0.15

The confidence intervals are for the difference in means comparing each dose group to the control. These were calculated by us based on the information available to us (mean and standard error estimates). Asterisks indicate statistically significant ($p<0.05$) difference in response compared to control group, as reported by the study authors. The information available to us differed from that available to the study authors, and statistical tests used varied by study; therefore there is not a one-to-one correspondence with the confidence intervals as calculated by us and the p-values reported by the study authors.

[a]mg/kg BW/day, unless otherwise specified. [b]40 mg/kg BW/day group not shown because fetal weight was not measured (all offspring deceased).

Table S47: 95% Confidence interval estimates for mammalian birth weight measurements, for each tested dose of PFOA (see Figure 5B).

Source [source ID]	Species	Route of Exposure	PFOA dose[a]	Lower bound	Upper bound
Hu et al. 2010 [68]	Mouse	Drinking water	0.5*	-0.23	0.05
			1*	-0.33	-0.01
Onishchenko et al. 2011 [3610]	Mouse	Food	0.3	-0.03	0.13
Yahia et al. 2010 [103]	Mouse	Gavage	1	0.00	0.06
			5*	-0.17	-0.09
			10*	-0.51	-0.43
Hines et al. 2009 [260]	Mouse	Gavage	0.01	-0.16	0.08
			0.1	-0.19	0.03
			0.3	-0.21	0.05
			1	-0.19	0.03
			5*	-0.30	-0.02
Fenton et al. 2009 [264]	Mouse	Gavage	0.1	-0.68	-0.04
			1	-0.24	0.20
			5	-0.27	0.23
White et al. 2009 [312]	Mouse	Gavage	5	-0.33	-0.01
Abbott et al. 2007 [528]	Mouse	Gavage	0.1	-0.04	0.14
			0.3	-0.11	0.13
			0.6	-0.10	0.08
			1	-0.12	0.06
White et al. 2007 [566]	Mouse	Gavage	5*	-0.21	-0.01
Wolf et al. 2007 [571b][b]	Mouse	Gavage	5*	-0.17	0.03
			20*	-0.34	-0.10
Wolf et al. 2007 [571a][b]	Mouse	Gavage	3	-0.14	0.02
			5*	-0.21	-0.07
Lau et al. 2006 [635]	Mouse	Gavage	1	-0.10	0.12
			3	-0.10	0.16
			5	-0.14	0.00
			10	-0.31	-0.11
			20*	-0.57	-0.17
White et al. 2011 [3862]	Mouse	Gavage	1	-0.11	0.01
			5*	-0.22	-0.08
Hinderliter et al. 2005 [711]	Rat	Gavage	3	-0.30	0.78
			10	-0.17	0.73
			30	-0.01	1.01
Staples et al. 1984 [1871]	Rat	Gavage	100	-0.51	0.31
York 2002 [5122]	Rat	Gavage	1*	-0.56	-0.06
			3	-0.30	0.24
			10	-0.34	0.14
			30*	-0.80	-0.26

Source [source ID]	Species	Route of Exposure	PFOA dose[a]	Lower bound	Upper bound
Staples et al. 1984 [1871]	Rat	Inhalation	0.1 mg/m^3	-0.21	0.61
			1 mg/m^3	-0.51	0.31
			10 mg/m^3	-0.70	0.30
			25 mg/m^3*	-1.06	-0.34

The confidence intervals are for the difference in means comparing each dose group to the control. These were calculated by us based on the information available to us (mean and standard error estimates). Asterisks indicate statistically significant ($p<0.05$) difference in response compared to control group, as reported by the study authors. The information available to us differed from that available to the study authors, and statistical tests used varied by study; therefore there is not a one-to-one correspondence with the confidence intervals as calculated by us and the p-values reported by the study authors.

[a]mg/kg BW/day, unless otherwise specified. [b]Study split into 2 datasets; a) cross foster (exposure GD1-17); b) windows of sensitivity (exposure groups GD7-17, GD10-17, GD13-17, GD15-17),

Table S48: 95% Confidence interval estimates for non-mammalian weight measurements, for each tested dose of PFOA (see Figure 6A).

Source [source ID]	Species	Route of Exposure	PFOA dose	Lower bound	Upper bound
Pinkas et al. 2010 [187]	Chicken	Egg Injection	5 mg/kg egg	-2.68	2.28
			10 mg/kg egg	-2.14	3.94
O'Brien et al. 2009 [236][a]	Chicken	Egg Injection	0.01 mg/kg egg	-1.50	1.72
			0.1 mg/kg egg	-0.26	3.20
			1 mg/kg egg	-0.80	2.22
			10 mg/kg egg	0.10	3.24
Jiang et al. 2012 [3926a][b]	Chicken	Egg Injection	0.5 mg/kg egg	-0.60	7.80
			1 mg/kg egg	-1.29	5.29
			2 mg/kg egg	-2.09	6.69
Jiang et al. 2012 [3926b][b]	Chicken	Egg Injection	0.5 mg/kg egg	-3.88	1.48
			1 mg/kg egg	-2.08	2.28
			2 mg/kg egg	-2.10	5.10
Wang et al. 2010 [86]	Fruit fly	Food	100 μM*	-1.64×10^{-5}	3.64×10^{-5}
			500 μM*	-2.27×10^{-4}	-1.53×10^{-4}
Spachmo and Arukwe 2012 [3932][a]	Salmon	Egg immersion	100 μg/L water	-0.07	0.01

The confidence intervals are for the difference in means comparing each dose group to the control. These were calculated by us based on the information available to us (mean and standard error estimates). Asterisks indicate statistically significant ($p<0.05$) difference in response compared to control group, as reported by the study authors. The information available to us differed from that available to the study authors, and statistical tests used varied by study; therefore there is not a one-to-one correspondence with the confidence intervals as calculated by us and the p-values reported by the study authors.

[a]Study did not test for statistical significance. [b]Study split into 2 datasets based on time of outcome measurement a) embryonic day 19; b) 16-24 hours post hatching.

Table S49: 95% Confidence interval estimates for non-mammalian length measurements, for each tested dose of PFOA (see Figure 6B).

Source [source ID]	Species	Route of Exposure	PFOA dose	Lower bound	Upper bound
Jiang et al. 2012 [3926]	Chicken	Egg injection	0.5 mg/kg egg	-0.77	1.23
			1 mg/kg egg	-0.71	1.27
			2 mg/kg egg	-1.28	0.42
Wang et al. 2010 [86]	Fruit fly	Food	100 μM*	-0.37	-0.04
			500 μM*	-0.66	-0.24
Spachmo and Arukwe 2012 [3932][a]	Salmon	Egg immersion	100 μg/L water	-0.09	0.17
Hagenaars et al. 2011 [59]	Zebrafish	Egg immersion	15 mg/L water	-0.08	0.20
			20 mg/L water	-0.09	0.21
			30 mg/L water*	-0.22	0.06
			40 mg/L water*	-0.21	0.07
			50 mg/L water*	-0.23	0.07
			75 mg/L water*	-0.30	0.00
			100 mg/L water*	-0.42	-0.14
			250 mg/L water*	-0.69	-0.43

The confidence intervals are for the difference in means comparing each dose group to the control. These were calculated by us based on the information available to us (mean and standard error estimates). Asterisks indicate statistically significant ($p<0.05$) difference in response compared to control group, as reported by the study authors. The information available to us differed from that available to the study authors, and statistical tests used varied by study; therefore there is not a one-to-one correspondence with the confidence intervals as calculated by us and the p-values reported by the study authors.

[a]Study did not test for statistical significance.

Instructions for making risk of bias determinations

1. Sequence generation

Was the allocation sequence adequately generated?

Criteria for a judgment of 'YES' (i.e. low risk of bias):
The investigators describe a random component in the sequence generation process such as:

- Referring to a random number table;
- Using a computer random number generator;
- Coin tossing;
- Shuffling cards or envelopes;
- Throwing dice;
- Drawing of lots.

Criteria for the judgment of 'PROBABLY YES' (i.e. probably low risk of bias):

There is insufficient information about the sequence generation process to permit a judgment of 'YES', but there is indirect evidence that suggests the sequence generation process was random, as described by the criteria for a judgment of 'YES'.

Criteria for the judgment of 'PROBABLY NO' (i.e. probably high risk of bias):

There is insufficient information about the sequence generation process to permit a judgment of 'NO', but there is indirect evidence that suggests a non-random component in the sequence generation process, as described by the criteria for a judgment of 'NO'.

Criteria for the judgment of 'NO' (i.e. high risk of bias):

The investigators describe a non-random component in the sequence generation process or that a random component was not used. Usually, the description would involve some systematic, non-random approach, for example:

- Sequence generated by date of birth;
- Sequence generated by some rule based on date (or day) of arrival at facility;
- Sequence generated by some rule based on record number.

Other non-random approaches happen much less frequently than the systematic approaches mentioned above and tend to be obvious. They usually involve judgment or some method of non-random categorization of animals, for example:

- Allocation by judgment of the investigator;
- Allocation by availability of the intervention.

Criteria for the judgment of 'NOT APPLICABLE' (risk of bias domain is not applicable to study):

There is evidence that sequence generation is not an element of study design capable of introducing risk of bias in the study.

2. Allocation concealment

Was allocation adequately concealed?

Criteria for a judgment of 'YES' (i.e. low risk of bias):

Investigators could not foresee assignment because one of the following, or an equivalent method, was used to conceal allocation:

- Sequentially numbered treatment containers of identical appearance to control; or
- Sequentially numbered prepared route of administration (e.g., pre-prepared water dosed with chemical) of identical appearance; or
- Sequentially numbered, opaque, sealed envelopes.

Criteria for the judgment of 'PROBABLY YES' (i.e. probably low risk of bias):

There is insufficient information about allocation concealment to permit a judgment of 'YES', but there is indirect evidence that suggests the allocation was adequately concealed, as described by the criteria for a judgment of 'YES'.

Criteria for the judgment of 'PROBABLY NO' (i.e. probably high risk of bias):

There is insufficient information about allocation concealment to permit a judgment of 'NO', but there is indirect evidence that suggests the allocation was not adequately concealed, as described by the criteria for a judgment of 'NO'.

Criteria for the judgment of 'NO' (i.e. high risk of bias):

Investigators handling experimental animals could possibly foresee assignments and thus introduce selection bias, such as allocation based on:

- Using an open random allocation schedule (e.g. a list of random numbers); or
- Assignment envelopes were used without appropriate safeguards (e.g. if envelopes were unsealed or non-opaque or not sequentially numbered); or
- Alternation or rotation; or
- Non-random and known criteria, such as date of birth; or
- Record number; or
- Any other explicitly unconcealed procedure.

Criteria for the judgment of 'NOT APPLICABLE' (risk of bias domain is not applicable to study):

There is evidence that allocation concealment is not an element of study design capable of introducing risk of bias in the study.

3. Blinding of personnel and outcome assessors

Was knowledge of the allocated interventions adequately prevented during the study?

Criteria for a judgment of 'YES' (i.e. low risk of bias):

Any one of the following:

- No blinding, but the review authors judge that the outcome and the outcome measurement are not likely to be influenced by lack of blinding; or
- Blinding of key study personnel ensured, and unlikely that the blinding could have been broken; or
- Some key study personnel were not blinded, but outcome assessment was blinded and the non-blinding of others unlikely to introduce bias.

Criteria for the judgment of 'PROBABLY YES' (i.e. probably low risk of bias):

There is insufficient information about blinding to permit a judgment of 'YES', but there is indirect evidence that suggests the study was adequately blinded, as described by the criteria for a judgment of 'YES'.

Criteria for the judgment of 'PROBABLY NO' (i.e. probably high risk of bias):

There is insufficient information about blinding to permit a judgment of 'NO', but there is indirect evidence that suggests the study was not adequately blinded, as described by the criteria for a judgment of 'NO'.

Criteria for the judgment of 'NO' (i.e. high risk of bias):

Any one of the following:

- No blinding or incomplete blinding, and the outcome or outcome measurement is likely to be influenced by lack of blinding; or
- Blinding of key study personnel attempted, but likely that the blinding could have been broken; or
- Some key study personnel were not blinded, and the non-blinding of others likely to introduce bias.

Criteria for the judgment of ‘NOT APPLICABLE’ (risk of bias domain is not applicable to study):

There is evidence that blinding is not an element of study design capable of introducing risk of bias in the study.

4. Incomplete outcome data

Were incomplete outcome data adequately addressed?

Criteria for a judgment of ‘YES’ (i.e. low risk of bias):

The number of animals assessed for outcome of interest is reported and data is provided indicating adequate follow up of all treated animals. Additional information provided by authors should be considered when making risk of bias judgments about incomplete outcome data. Additionally, any one of the following:

- No missing outcome data; or
- Reasons for missing outcome data unlikely to be related to true outcome (for survival data, censoring unlikely to be introducing bias); or
- Missing outcome data is provided and is balanced in numbers across intervention groups, with similar reasons for missing data across groups; or
- For dichotomous outcome data, the proportion of missing outcomes compared with observed event risk not enough to have a biologically relevant impact on the intervention effect estimate; or
- For continuous outcome data, plausible effect size (difference in means or standardized difference in means) among missing outcomes not enough to have a biologically relevant impact on observed effect size; or
- Missing data have been imputed using appropriate statistical methods.

Criteria for the judgment of ‘PROBABLY YES’ (i.e. probably low risk of bias):

There is insufficient information about incomplete outcome data to permit a judgment of ‘YES’, but there is indirect evidence that suggests incomplete outcome data were adequately addressed, as described by the criteria for a judgment of ‘YES’.

Criteria for the judgment of ‘PROBABLY NO’ (i.e. probably high risk of bias):

There is insufficient information about incomplete outcome data to permit a judgment of ‘NO’, but there is indirect evidence that suggests incomplete outcome data was not adequately addressed, as described by the criteria for a judgment of ‘NO’.

Criteria for the judgment of ‘NO’ (i.e. high risk of bias):

The number of animals allocated not reported and no data is provided to indicate that there was adequate follow up of all treated animals. Additionally, any one of the following:

- Reason for missing outcome data likely to be related to true outcome, with either imbalance in numbers or reasons for missing data across intervention groups; or
- For dichotomous outcome data, the proportion of missing outcomes compared with observed event risk enough to induce biologically relevant bias in intervention effect estimate; or
- For continuous outcome data, plausible effect size (difference in means or standardized difference in means) among missing outcomes enough to induce biologically relevant bias in observed effect size; or
- ‘As-treated’ analysis done with substantial departure of the intervention received from that assigned at randomization; or
- Potentially inappropriate application of simple imputation.

Criteria for the judgment of ‘NOT APPLICABLE’ (risk of bias domain is not applicable to study):

There is evidence that incomplete outcome data is not capable of introducing risk of bias in the study.

5. Selective outcome reporting

Are reports of the study free of suggestion of selective outcome reporting?

Criteria for a judgment of ‘YES’ (i.e. low risk of bias):

All of the study’s pre-specified (primary and secondary) outcomes outlined in the methods, abstract, and/or introduction that are of interest in the review have been reported in the pre-specified way, including the number of animals analyzed for outcomes of interest. Additional information provided by authors should be considered when making risk of bias judgments for selective outcome reporting.

Criteria for the judgment of ‘PROBABLY YES’ (i.e. probably low risk of bias):

There is insufficient information about selective outcome reporting to permit a judgment of ‘YES’, but there is indirect evidence that suggests the study was free of selective reporting, as described by the criteria for a judgment of ‘YES’.

Criteria for the judgment of ‘PROBABLY NO’ (i.e. probably high risk of bias):

There is insufficient information about selective outcome reporting to permit a judgment of 'NO', but there is indirect evidence that suggests the study was not free of selective reporting, as described by the criteria for a judgment of 'NO'.

Criteria for the judgment of 'NO' (i.e. high risk of bias):

The study was not free of selective reporting. The following should be considered:

- Authors did not report numbers analyzed for outcomes of interest; or
- Not all of the study's pre-specified primary outcomes (as outlined in the protocol, title, abstract, and/or introduction) that are of interest in the review have been reported; or
- One or more primary outcomes is reported using measurements, analysis methods or subsets of the data (e.g. subscales) that were not pre-specified (unless clear justification for their reporting is provided, such as an unexpected adverse effect); or
- One or more reported primary outcomes were not pre-specified (unless clear justification for their reporting is provided, such as an unexpected adverse effect); or
- One or more outcomes of interest in the review are reported incompletely so that they cannot be entered in a meta-analysis; or
- The study report fails to include results for a key outcome that would be expected to have been reported for such a study.

Criteria for the judgment of 'NOT APPLICABLE' (risk of bias domain is not applicable to study):

There is evidence that selective outcome reporting is not capable of introducing risk of bias in the study.

6. Other potential threats to validity

Was the study apparently free of other problems that could put it at a risk of bias?

Criteria for a judgment of 'YES' (i.e. low risk of bias):

The study appears to be free of other sources of bias.

Criteria for the judgment of 'PROBABLY YES' (i.e. probably low risk of bias):

There is insufficient information to permit a judgment of 'YES', but there is indirect evidence that suggests the study was free of other threats to validity, as described by the criteria for a judgment of 'YES'.

Criteria for the judgment of ‘PROBABLY NO’ (i.e. probably high risk of bias):

There is insufficient information to permit a judgment of ‘NO’, but there is indirect evidence that suggests the study was not free of other threats to validity, as described by the criteria for a judgment of ‘NO’.

Criteria for the judgment of ‘NO’ (i.e. high risk of bias):

There is at least one important risk of bias. For example, the study:

- Had a potential source of bias related to the specific study design used;
- Stopped early due to some data-dependent process (including a formal-stopping rule);
- Had extreme baseline imbalance (improper control group);
- Has been claimed to have been fraudulent;
- The conduct of the study is affected by interim results (e.g. recruiting additional animals from a subgroup showing more benefit);
- There is deviation from the study protocol in a way that does not reflect typical practice (e.g. post hoc stepping-up of doses to exaggerated levels);
- There is pre-randomization administration of an intervention that could enhance or diminish the effect of a subsequent, randomized, intervention; inappropriate administration of an intervention (or co-intervention);
- Occurrence of ‘null bias’ due to interventions being insufficiently well delivered or overly wide inclusion criteria for animals;
- An insensitive instrument is used to measure outcomes (which can lead to under-estimation of both beneficial and harmful effects);
- Selective reporting of subgroups;
- Had some other problem.

Criteria for the judgment of ‘NOT APPLICABLE’ (risk of bias domain is not applicable to study):

There is evidence that other potential threats to validity are not capable of introducing risk of bias in the study.

7. Conflict of interest

Was the study free of support from a company, study author, or other entity having a financial interest in any of the treatments studied?

Criteria for a judgment of ‘YES’ (i.e. low risk of bias):

The study did not receive support from a company, study author, or other entity having a financial interest in the outcome of the study. A conflict of interest statement is provided to indicate the authors have no financial interest and there is evidence of the entities not having a financial interest. Examples of this evidence include the following:

- Funding source is limited to government, non-profit organizations, or academic grants funded by government, foundations and/or non-profit organizations;
- Chemicals or other treatment used in study were purchased from a supplier;
- Company affiliated staff are not mentioned in the acknowledgements section;
- Authors were not employees of a company with a financial interest in the outcome of the study;
- Company with a financial interest in the outcome of the study was not involved in the design, conduct, analysis, or reporting of the study and authors had complete access to the data;
- Study authors make a claim denying conflicts of interest;
- Study authors are unaffiliated with companies with financial interest, and there is no reason to believe a conflict of interest exists;
- All study authors are affiliated with a government agency (are prohibited from involvement in projects for which there is a conflict of interest or an appearance of conflict of interest).

Criteria for the judgment of 'PROBABLY YES' (i.e. probably low risk of bias):

There is insufficient information to permit a judgment of 'YES', for example there is no conflict of interest statement denying financial interests, but there is evidence that suggests the study was free of support from a company, study author, or other entity having a financial interest in the outcome of the study, as described by the criteria for a judgment of 'YES'.

Criteria for the judgment of 'PROBABLY NO' (i.e. probably high risk of bias):

There is insufficient information to permit a judgment of 'NO', but there is indirect evidence that suggests the study was not free of support from a company, study author, or other entity having a financial interest in the outcome of the study, as described by the criteria for a judgment of 'NO'.

Criteria for the judgment of 'NO' (i.e. high risk of bias):

The study received support from a company, study author, or other entity having a financial interest in the outcome of the study. Examples of support include:

- Research funds;
- Writing services;
- Author/staff from study was employee or otherwise affiliated with company with financial interest;

- Company limited author access to the data;
- Company was involved in the design, conduct, analysis, or reporting of the study;
- Study authors claim a conflict of interest.

Criteria for the judgment of 'NOT APPLICABLE' (risk of bias domain is not applicable to study):

There is evidence that conflicts of interest are not capable of introducing risk of bias in the study.

References

Abbott BD, Wolf CJ, Schmid JE, Das KP, Zehr RD, Helfant L, et al. 2007. Perfluorooctanoic acid induced developmental toxicity in the mouse is dependent on expression of peroxisome proliferator activated receptor-alpha. Toxicol Sci 98:571-581.

ATSDR (Agency for Toxic Substances & Disease Registry). 2012. Interaction Profiles for Toxic Substances. Available: http://www.atsdr.cdc.gov/interactionprofiles/index.asp [accessed 6 February 2012].

ATSDR (Agency for Toxic Substances & Disease Registry). 2012. Toxic Substances Portal. Available: http://www.atsdr.cdc.gov/toxprofiles/index.asp [accessed 6 February 2012].

Boberg J, Metzdorff S, Wortziger R, Axelstad M, Brokken L, Vinggaard AM, et al. 2008. Impact of diisobutyl phthalate and other PPAR agonists on steroidogenesis and plasma insulin and leptin levels in fetal rats. Toxicology 250:75-81.

Butenhoff JL, Kennedy GL, Jr., Frame SR, O'Connor JC, York RG. 2004. The reproductive toxicology of ammonium perfluorooctanoate (APFO) in the rat. Toxicology 196:95-116.

CalEPA (California Environmental Protection Agency). 2012. Office of Environmental Health Hazard Assessment Risk Assessment. Available: http://www.oehha.ca.gov/risk.html [accessed 6 February 2012].

European Chemicals Agency. 2012. Homepage. Available: http://echa.europa.eu/web/guest/home [accessed 6 February 2012].

Fenton SE, Reiner JL, Nakayama SF, Delinsky AD, Stanko JP, Hines EP, et al. 2009. Analysis of PFOA in dosed CD-1 mice. Part 2. Disposition of PFOA in tissues and fluids from pregnant and lactating mice and their pups. Reprod Toxicol 27:365-372.

Hagenaars A, Vergauwen L, De Coen W, Knapen D. 2011. Structure-activity relationship assessment of four perfluorinated chemicals using a prolonged zebrafish early life stage test. Chemosphere 82:764-772.

Health Canada. 2012. First Priority Substances List (PSL1) Assessments. Available: http://www.hc-sc.gc.ca/ewh-semt/pubs/contaminants/psl1-lsp1/index-eng.php [accessed 3 February 2012].

Health Canada. 2012. Second Priority Substances List (PSL2) Assessments. Available: http://www.hc-sc.gc.ca/ewh-semt/pubs/contaminants/psl2-lsp2/index-eng.php [accessed 3 February 2012].

Hinderliter PM, Mylchreest E, Gannon SA, Butenhoff JL, Kennedy GL Jr. 2005. Perfluorooctanoate: Placental and lactational transport pharmacokinetics in rats. Toxicology 211:139-148.

Hines EP, White SS, Stanko JP, Gibbs-Flournoy EA, Lau C, Fenton SE. 2009. Phenotypic dichotomy following developmental exposure to perfluorooctanoic acid (PFOA) in female CD-1 mice: Low doses induce elevated serum leptin and insulin, and overweight in mid-life. Mol Cell Endocrinol 304:97-105.

Hooijmans CR, Tillema A, Leenaars M, Ritskes-Hoitinga M. 2010b. Enhancing search efficiency by means of a search filter for finding all studies on animal experimentation in PubMed. Lab Anim 44:170-175.

Hu Q, Strynar MJ, DeWitt JC. 2010. Are developmentally exposed C57BL/6 mice insensitive to suppression of TDAR by PFOA? J Immunotoxicol 7:344-349.

IARC (International Agency for Research on Cancer). 2012. Monographs on the Evaluation of Carcinogenic Risks to Humans. Available: http://monographs.iarc.fr/ENG/Monographs/PDFs/index.php [accessed 3 February 2012].

ILSI (International Life Sciences Institute). 2012. Homepage. Available: http://www.ilsi.org/Pages/HomePage.aspx [accessed 3 February 2012].

IPCS (International Programme on Chemical Safety). 2012. INCHEM. Available: http://www.inchem.org/ [accessed 3 February 2012].

Jiang Q, Lust RM, Strynar MJ, Dagnino S, DeWitt JC. 2012. Perflurooctanoic acid induces developmental cardiotoxicity in chicken embryos and hatchlings. Toxicology 293:97-106.

Lau C, Thibodeaux JR, Hanson RG, Narotsky MG, Rogers JM, Lindstrom AB, et al. 2006. Effects of perfluorooctanoic acid exposure during pregnancy in the mouse. Toxicol Sci 90:510-518.

National Center for Biotechnology Information. 2012. PubMed. Available: http://www.ncbi.nlm.nih.gov/pubmed/ [accessed 3 February 2012].

National Library of Medicine. 2012. Chemical Carcinogenesis Research Information System (CCRIS). Available: http://toxnet.nlm.nih.gov/cgi-bin/sis/htmlgen?CCRIS [accessed 6 February 2012].

National Library of Medicine. 2012. ChemIDplus Advanced. Available: http://chem.sis.nlm.nih.gov/chemidplus/ [accessed 6 February 2012].

National Library of Medicine. 2012. Developmental and Reproductive Toxicology Database (DART). Available: http://toxnet.nlm.nih.gov/cgi-bin/sis/htmlgen?DARTETIC [accessed 6 February 2012].

National Library of Medicine. 2012. Environmental Mutagen Information Center (EMIC). Available: http://toxnet.nlm.nih.gov/ [accessed 6 February 2012].

National Library of Medicine. 2012. Genetic Toxicology Data Bank (GENE-TOX). Available: http://toxnet.nlm.nih.gov/cgi-bin/sis/htmlgen?GENETOX [accessed 6 February 2012].

National Library of Medicine. 2012. Hazardous Substances Data Bank (HSDB). Available: http://toxnet.nlm.nih.gov/cgi-bin/sis/htmlgen?HSDB [accessed 6 February 2012].

National Library of Medicine. 2012. Toxicology Data Network (TOXNET). Available: http://toxnet.nlm.nih.gov/ [accessed 6 February 2012].

National Library of Medicine. 2012. Toxicology Literature Online (TOXLINE). Available: http://toxnet.nlm.nih.gov/cgi-bin/sis/htmlgen?TOXLINE [accessed 6 February 2012].

NIOSH (National Institute for Occupational Safety and Health). 2012. NIOSHTIC-2. Available: https://www2a.cdc.gov/nioshtic-2/advsearch2.asp [accessed 6 February 2012].

NIOSH (National Institute for Occupational Safety and Health). 2012. Registry of Toxic Effects of Chemical Substances (RTECS). Available: http://www.cdc.gov/niosh/rtecs/default.html [accessed 6 February 2012].

NTP (National Toxicology Program). 2012. Management Status Report. Available: http://ntp.niehs.nih.gov/go/MSR-index [accessed 6 February 2012].

NTP (National Toxicology Program). 2012. Program Results and Status Search. Available: http://ntp.niehs.nih.gov/?objectid=720160DB-BDB7-CEBA-F7CC2DE0A230C920 [accessed 6 February 2012].

NTP (National Toxicology Program). 2012. Program Report on Carcinogens. Available: http://ntp.niehs.nih.gov/?objectid=72016262-BDB7-CEBA-FA60E922B18C2540 [accessed 6 February 2012].

O'Brien JM, Crump D, Mundy LJ, Chu S, McLaren KK, Vongphachan V, et al. 2009. Pipping success and liver mRNA expression in chicken embryos exposed in ovo to C8 and C11 perfluorinated carboxylic acids and C10 perfluorinated sulfonate. Toxicol Lett 190:134-139.

Onishchenko N, Fischer C, Wan Ibrahim WN, Negri S, Spulber S, Cottica D, et al. 2011. Prenatal exposure to PFOS or PFOA alters motor function in mice in a sex-related manner. Neurotox Res 19:452-461.

Pinkas A, Slotkin TA, Brick-Turin Y, Van der Zee EA, Yanai J. 2010. Neurobehavioral teratogenicity of perfluorinated alkyls in an avian model. Neurotoxicol Teratol 32:182-186.

Royal Society of Chemistry. 2012. ChemSpider. Available: http://www.chemspider.com/ [accessed 6 February 2012].

Spachmo B, Arukwe A. 2012. Endocrine and developmental effects in Atlantic salmon (Salmo salar) exposed to perfluorooctane sulfonic or perfluorooctane carboxylic acids. Aquat Toxicol 108:112-124.

Staples RE, Burgess BA, Kerns WD. 1984. The embryo-fetal toxicity and teratogenic potential of ammonium perfluorooctanoate (APFO) in the rat. Fundam Appl Toxicol 4:429-440.

Thompson Reuters. 2012. Web of Science. Available: http://apps.webofknowledge.com/WOS_GeneralSearch_input.do?product=WOS&search_mode=GeneralSearch&SID=3BIcWf14OmZnOOyxRJ9&preferencesSaved= [accessed 3 February 2012].

Toxicology Excellence for Risk Assessment. 2012. Homepage. Available: http://www.tera.org/ [accessed 3 February 2012].

Toxicology Excellence for Risk Assessment. 2012. International Toxicity Estimates for Risk (ITER). Available: http://iter.ctcnet.net/publicurl/pub_search_list.cfm [accessed 3 February 2012].

US EPA (U.S. Environmental Protection Agency). 2012. Acute Exposure Guideline Levels Chemicals. Available: http://www.epa.gov/oppt/aegl/pubs/chemlist.htm [accessed 6 February 2012].

US EPA (U.S. Environmental Protection Agency). 2012. EPA Science Inventory. Available: http://cfpub.epa.gov/si/ [accessed 6 February 2012].

US EPA (U.S. Environmental Protection Agency). 2012. Health & Environmental Research Online (HERO). Available: http://hero.epa.gov/ [accessed 6 February 2012].

US EPA (U.S. Environmental Protection Agency). 2012. Integrated Risk Information System (IRIS). Available: http://www.epa.gov/iris/ [accessed 6 February 2012].

US EPA (U.S. Environmental Protection Agency). 2012. National Environmental Publications Internet Site (NEPIS). Available: http://nepis.epa.gov [accessed 6 February 2012].

US EPA (U.S. Environmental Protection Agency). 2012. National Service Center for Environmental Publications (NSCEP). Available: http://www.epa.gov/ncepihom/ [accessed 6 February 2012].

US EPA (U.S. Environmental Protection Agency). 2012. Substance Registry Services. Available: http://www.epa.gov/srs/ [accessed 6 February 2012].

US EPA (U.S. Environmental Protection Agency). 2012. Toxic Substances Control Act Test Submissions (TSCATS). Available: http://www.ntis.gov/products/ots.aspx [accessed 6 February 2012].

Wang J, Li Y, Liu Y, Zhang H, Dai J. 2010. Disturbance of perfluorooctanoic acid on development and behavior in Drosophila larvae. Environ Toxicol Chem 29:2117-2122.

White SS, Calafat AM, Kuklenyik Z, Villanueva L, Zehr RD, Helfant L, et al. 2007. Gestational PFOA exposure of mice is associated with altered mammary gland development in dams and female offspring. Toxicol Sci 96:133-144.

White SS, Kato K, Jia LT, Basden BJ, Calafat AM, Hines EP, et al. 2009. Effects of perfluorooctanoic acid on mouse mammary gland development and differentiation resulting from cross-foster and restricted gestational exposures. Reprod Toxicol 27:289-298.

White SS, Stanko JP, Kato K, Calafat AM, Hines EP, Fenton SE. 2011. Gestational and chronic low-dose PFOA exposures and mammary gland growth and differentiation in three generations of CD-1 mice. Environ Health Perspect 119:1070-1076.

WHO (World Health Organization). 2012. Concise International Chemical Assessment Documents. Available: http://www.who.int/ipcs/publications/cicad/en/index.html [accessed 6 February 2012].

WHO (World Health Organization). 2012. Environmental Health Criteria. Available: http://www.who.int/ipcs/publications/ehc/en/index.html [accessed 6 February 2012].

Wolf CJ, Fenton SE, Schmid JE, Calafat AM, Kuklenyik Z, Bryant XA, et al. 2007. Developmental toxicity of perfluorooctanoic acid in the CD-1 mouse after cross-foster and restricted gestational exposures. Toxicol Sci 95:462-473.

Yahia D, El-Nasser MA, Abedel-Latif M, Tsukuba C, Yoshida M, Sato I, et al. 2010. Effects of perfluorooctanoic acid (PFOA) exposure to pregnant mice on reproduction. J Toxicol Sci 35:527-533.

York R. 2002. Oral (gavage) two-generation (one litter per generation) reproduction study of ammonium perfluorooctanoate (APFO) in rats. Report prepared for 3M, St. Paul, MN. Sponsor's Study No T-6889.6. Reviewed in US EPA AR226-1092. Horsham, PA:Argus Research.
